# Why Do Dietary Saponins Produce Inconsistent Responses in Ruminants? A Review

**DOI:** 10.3390/cimb48101057

**Published:** 2026-10-14

**Authors:** Maghsoud Besharati, Sina Salimolnafs, Deniz Azhir

**Affiliations:** 1 Department of Animal Science, Ahar Faculty of Agriculture and Natural Resources, University of Tabriz, Tabriz 51666, Iran; sina.salimolnafs@studio.unibo.it; 2 Department of Food Science and Technology, Faculty of Agriculture, University of Tabriz, Tabriz 51666, Iran; d.azhir@hotmail.com

**Keywords:** saponins, ruminants, rumen fermentation, methane mitigation, phytogenic feed additives

## Abstract

Saponins are structurally diverse plant-derived glycosides that have attracted increasing interest as phytogenic feed additives for improving the efficiency and sustainability of ruminant production. This narrative review critically examines the structural characteristics, biological activities, and nutritional applications of dietary saponins, with particular emphasis on the links between molecular structure, rumen microbial modulation, and animal responses. Saponins are broadly classified as steroidal or triterpenoid compounds, and differences in their aglycone structure, glycosylation pattern, and botanical origin contribute substantially to their biological activity. Evidence from in vitro and in vivo studies indicates that dietary saponins can alter rumen microbial populations, particularly protozoa, and consequently influence volatile fatty acid production, ammonia metabolism, microbial protein synthesis, nutrient utilization, and enteric methane emissions. Their potential benefits extend beyond ruminal fermentation to animal health, antioxidant and metabolic responses, reproductive function, and milk and meat quality. However, responses are inconsistent across studies and are strongly influenced by saponin source, chemical composition, dose, basal diet, animal species, physiological status, and microbial adaptation. Recent evidence suggests that moderate supplementation can provide beneficial effects without compromising feed intake, whereas excessive inclusion may impair fiber degradation and nutrient digestibility. The review therefore emphasizes that saponin efficacy should not be evaluated solely on total concentration but in relation to chemical structure and feeding conditions. Finally, major knowledge gaps are identified, including the need for standardized saponin preparations, species-specific supplementation strategies, improved understanding of microbial adaptation and structure–activity relationships, and long-term validation under commercial production conditions.

## 1. Introduction

The worldwide demand for animal-derived foods is steadily rising, thereby exerting pressure on ruminant production systems to enhance efficiency without compromising animal health or product quality [1]. Concurrently, growing concerns regarding antimicrobial resistance and food safety have prompted numerous countries to limit or prohibit the prophylactic use of antibiotic growth promoters [2,3,4]. This regulatory shift has increased interest in phytogenic feed additives as natural alternatives to promote both productivity and animal health [1,5,6].

Historically, antibiotic growth promoters were widely used to enhance animal performance and feed efficiency [2,3,4]. However, growing concerns about antimicrobial resistance have prompted regulatory action [3,4]. The presence of drug residues in animal-derived products has further accelerated these measures [3,4]. These concerns culminated in the European Union’s comprehensive ban on antibiotic growth promoters in 2006 [2,3,4]. Consequently, research has increasingly focused on natural alternatives [5,6]. Phytogenic feed additives derived from plants, herbs, and essential oils are now primary candidates for sustaining animal health and productivity without relying on antibiotics [1,5,6].

Natural products, especially phytochemicals, have historically constituted a significant reservoir of bioactive compounds due to their extensive chemical diversity and biological significance. These substances have garnered substantial attention within animal nutrition research for their potential to improve health and productivity via various biological pathways [5,6]. Among phytochemicals, saponins are one of the most extensively studied groups. These naturally occurring glycosides are found in both medicinal and edible plants and exhibit a broad spectrum of biological activities relevant to animal nutrition, including antimicrobial, immunomodulatory, and hypocholesterolemic effects [7,8]. Steroidal saponins in plants are typically derived from *Polygonatum*, *Ophiopogon*, and *Agave*, while triterpenoid saponins are commonly found in *Panax ginseng*, *Panax notoginseng*, and *Glycyrrhiza*.

As small-molecule natural surfactants with high surface activity, saponins are currently used as food emulsifiers, primarily extracted from *Sapindus* (soapnut), *Camellia* (tea seed), and soybean sources [9,10]. Their amphiphilic nature, biocompatibility, and biodegradability have further expanded their applications in cosmetics, pharmaceuticals, food formulation, and environmental remediation [11,12,13].

In animal nutrition, saponins have attracted growing attention for their ability to modulate rumen fermentation, enhance nutrient utilization, and improve animal health and productivity. Particular attention has been directed toward the antiprotozoal, methane-mitigating, antioxidant, and immunomodulatory properties of saponins, which may contribute to improved feed efficiency and sustainability of livestock production systems. Moreover, the rapid discovery of new steroidal and triterpenoid saponins has highlighted the need for an updated and integrated assessment of their structural diversity and biological significance in the context of ruminant nutrition.

Despite extensive studies on the chemistry and bioactivities of saponins, inconsistent findings regarding their effects on rumen fermentation, methane mitigation, nutrient utilization, and animal productivity continue to limit their practical application in livestock production systems. This inconsistency raises three critical questions not systematically addressed in previous reviews: (1) To what extent do structural features, aglycone type (steroidal vs. triterpenoid), and glycosylation pattern (monodesmosidic vs. bidesmosidic) determine rumen fermentation responses and methanogenesis inhibition? (2) Is there a universal dose-dependent trade-off between methane suppression and fibrolytic activity across all saponin sources, or does this relationship vary with botanical origin and ruminant species? (3) Can rumen microbiota permanently adapt to saponins during prolonged feeding, and if so, what strategies (e.g., rotational supplementation or combinations with tannins) could sustain long-term efficacy? We hypothesize that while all saponins exert membrane-interacting effects, triterpenoid saponins with monodesmosidic structures have more potent but less selective antimicrobial activity than steroidal types, and that moderate inclusion levels (≤40 g/kg DM) consistently improve nitrogen utilization without compromising fiber digestion, provided gradual adaptation prevents permanent microbial resilience. Therefore, this review aims not only to provide a comprehensive overview of saponin classification, structure, and bioactivities but also to critically evaluate the evidence supporting or refuting these hypotheses, with particular emphasis on the relationships between chemical structure, rumen modulation, animal performance, methane mitigation, and sustainability, while identifying key research gaps for precision application in commercial ruminant systems.

## 2. Review Design

This manuscript was developed as a structured critical review rather than a systematic review or meta-analysis. The primary objective was to critically synthesize the available evidence on dietary saponins in ruminant nutrition and, in particular, to explain the substantial variability observed among studies in relation to ruminal fermentation, methane production, nutrient utilization, animal performance, product quality, health, and microbial adaptation. The review therefore focused not only on whether saponins affect a given response, but also on the factors that may explain differences among experimental findings.

### 2.1. Literature Search

The literature search was conducted using Scopus, Web of Science Core Collection, PubMed, and CAB Abstracts. Google Scholar was used as a supplementary source to identify potentially relevant publications and to check the reference lists of key reviews and highly relevant primary studies. The search included publications available up to October 2025, without imposing an a priori restriction on publication year.

The search strategy was constructed around three major concepts: (1) saponins, (2) ruminant species, and (3) nutritional, ruminal, productive, metabolic, and health-related responses. Representative search terms included “saponin,” “steroidal saponin,” “triterpenoid saponin,” “tea saponin,” “Yucca saponin,” “Quillaja saponin,” “alfalfa saponin,” “ruminant,” “cattle,” “cow,” “dairy cow,” “beef cattle,” “sheep,” “lamb,” “goat,” and “buffalo.” These terms were combined with outcome-related terms including “rumen fermentation,” “methane,” “methanogenesis,” “protozoa,” “nutrient digestibility,” “feed intake,” “microbial protein,” “nitrogen utilization,” “animal performance,” “milk,” “meat,” “health,” “antioxidant,” and “reproduction.” Search syntax was adapted to the requirements of individual databases.

In addition to database searching, the reference lists of key reviews and highly relevant primary studies were examined to identify additional publications that might not have been retrieved through the electronic database searches. This backward citation searching was used as a supplementary identification strategy rather than as a substitute for the database search.

### 2.2. Eligibility and Study Selection

Studies were considered eligible for the core evidence base when they provided experimental or mechanistic evidence directly relevant to dietary saponins or saponin-containing feed materials in ruminants. The selection process was based on relevance to the objectives of the review rather than on the manuscript subsection in which a study could subsequently be discussed.

Priority was given to in vivo feeding studies in cattle, sheep, goats, and buffalo because these studies provide direct evidence of animal-level responses. Well-designed in vitro rumen fermentation studies, batch-culture experiments, and rumen simulation studies were also considered when they provided mechanistic information relevant to ruminal fermentation, microbial populations, methanogenesis, nutrient degradation, or microbial adaptation. Studies involving non-ruminant species or humans were not considered primary evidence for nutritional conclusions and were used only when limited background information was required to explain saponin chemistry or a biological mechanism directly relevant to ruminant nutrition.

Studies were excluded from the core evidence base when they:

(i) investigated non-ruminant species without direct mechanistic relevance to ruminant nutrition;
(ii) focused exclusively on pharmaceutical, anticancer, neuroprotective, or other unrelated applications of saponins;
(iii) did not evaluate dietary saponins or a saponin-containing feed material in a context relevant to ruminant nutrition;
(iv) provided insufficient information to interpret the relevance of the reported findings to the objectives of the review.

Because this was a structured critical review, study selection was not based solely on a formal risk-of-bias scoring system. Instead, the relevance and evidentiary contribution of each study were critically considered according to experimental design, animal species, sample size, saponin source, chemical characterization, supplementation level, basal diet, duration of exposure, analytical methods, and outcomes measured.

### 2.3. Evidence Domains and Organization of the Review

After identification and eligibility assessment, the selected literature was organized into predefined evidence domains. These included:

1. saponin sources, chemical structure, and structure–activity relationships;
2. ruminal degradation and microbial interactions;
3. rumen fermentation and microbial ecology;
4. enteric methane production;
5. feed intake, nutrient digestibility, and nutrient utilization;
6. microbial protein synthesis and nitrogen metabolism;
7. growth, feed efficiency, milk and meat production;
8. metabolic, antioxidant, immune, and reproductive responses;
9. microbial adaptation and the persistence of saponin effects during prolonged supplementation.

Importantly, these domains were used to organize and synthesize the evidence after study identification, rather than to determine which studies were searched for or selected. A single study could therefore contribute evidence to more than one domain when its experimental outcomes were relevant to multiple aspects of the review question.

Particular attention was given to differences between in vitro and in vivo evidence, as well as to dose-dependent responses, saponin source and chemical structure, duration of supplementation, basal diet, animal species, physiological status, and experimental conditions. Studies reporting apparently contradictory results were examined together to identify plausible explanations for the observed heterogeneity.

### 2.4. Evidence Synthesis

The evidence was synthesized qualitatively because substantial heterogeneity existed among studies in terms of saponin sources, chemical composition, supplementation doses, animal species, diets, experimental durations, and outcome variables. Quantitative pooling was therefore not attempted. Recent systematic reviews and meta-analyses were used as secondary evidence to identify major trends and knowledge gaps, whereas conclusions concerning specific biological responses were primarily based on relevant primary experimental studies.

The overall synthesis was structured around the conceptual pathway:

Saponin source and chemical structure → ruminal microbial interactions → fermentation and methane responses → nutrient utilization → animal performance and product quality → systemic responses and microbial adaptation.

This framework was used to maintain a direct connection between the literature-selection strategy and the principal objective of the review. It also enabled apparently inconsistent findings to be interpreted in relation to biological and experimental moderators rather than being presented as isolated observations.

Because the original literature search was conducted as a structured critical review rather than as a prospectively registered systematic review, numerical database-hit counts were not recorded in a standardized PRISMA database log. We therefore do not retrospectively estimate or reconstruct the number of records retrieved from individual databases. Instead, the revised manuscript provides the databases searched, search concepts, search period, eligibility criteria, supplementary citation-search procedure, and evidence domains used for synthesis, thereby providing a transparent description of the literature-selection methodology without introducing unsupported numerical estimates.

### 2.5. Distinction Between Purified Saponins, Characterized Extracts, Whole Plants, and Mixtures

Throughout this review, the term “saponin” is used to refer to a broad class of glycosidic compounds, but the biological effects reported in the literature depend critically on the nature of the test material. Four categories of test materials must be distinguished:

Purified saponins: Individual saponin compounds or fractions of high purity (e.g., >95%) that have been isolated and chemically characterized. These materials allow the most direct attribution of effects to saponins.

Characterized saponin extracts: Extracts in which the total saponin content has been quantified and the aglycone type or glycosylation pattern has been at least partially characterized. Effects can be reasonably attributed to saponins, but the presence of other co-extracted phytochemicals should be considered.

Whole plants or plant parts: Plant materials (e.g., alfalfa meal, tea seed cake) that contain saponins along with other phytochemicals. Effects cannot be attributed solely to saponins.

Mixtures containing other phytochemicals: Formulations that combine saponins with tannins, essential oils, flavonoids, or other bioactive compounds. The observed effects may result from additive, synergistic, or antagonistic interactions.

### 2.6. Generative AI-Assisted Figure Preparation

**Figure 2:**
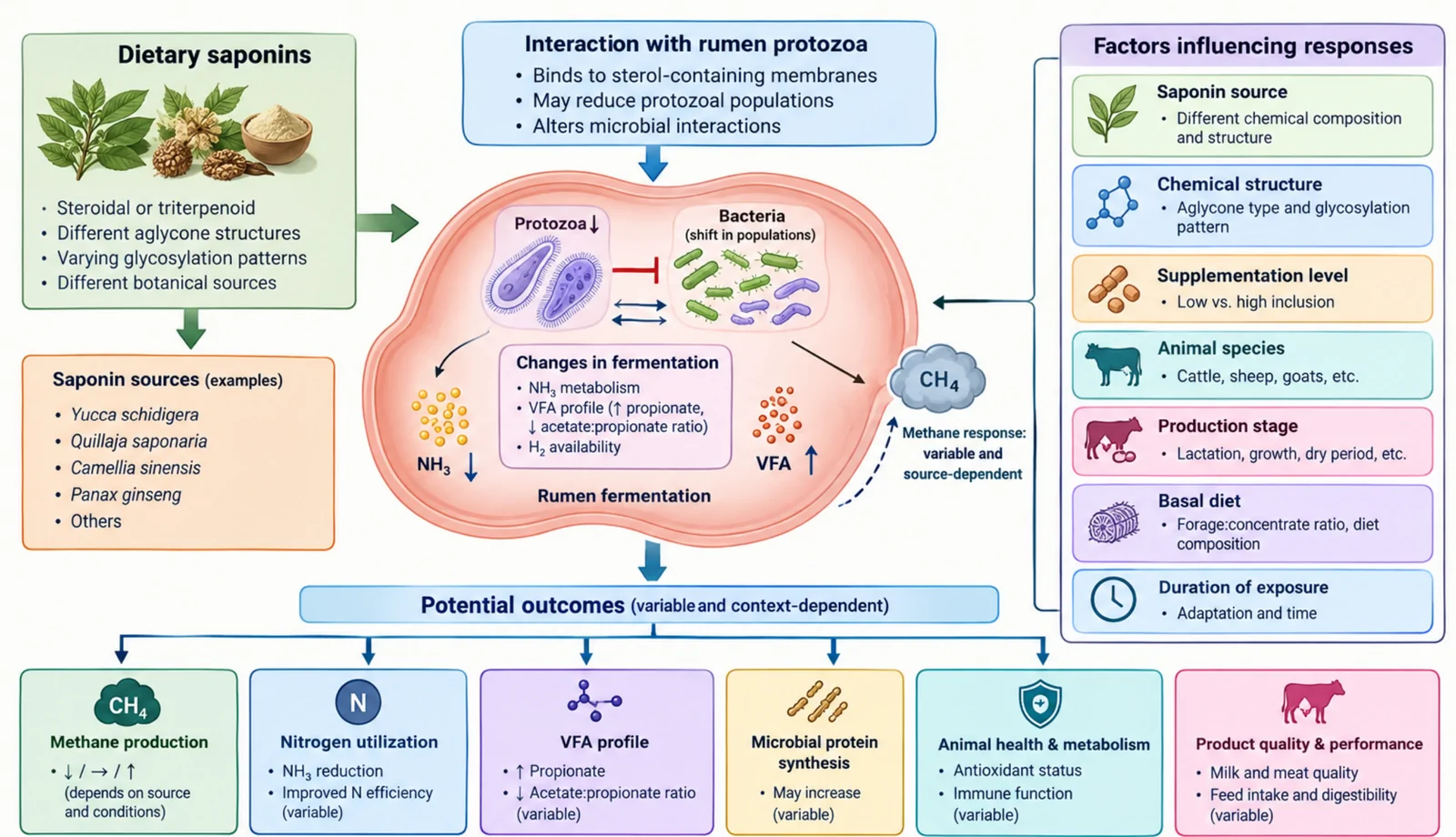
Conceptual overview of the potential mechanisms by which dietary saponins may influence rumen fermentation and related responses. Saponins can interact with sterol-containing protozoal membranes and may reduce protozoal populations, alter microbial interactions, and subsequently influence ammonia metabolism, volatile fatty acid (VFA) profiles, hydrogen availability, and methane production. These effects are not uniform and may differ according to saponin source, chemical structure, supplementation level, animal species, production stage, basal diet, and duration of exposure, as well as microbial adaptation. Accordingly, the pathways illustrated represent potential mechanisms rather than universal responses across ruminants. The figure was prepared with assistance from the web-based version of Google NotebookLM and was subsequently reviewed and verified by the authors for scientific accuracy and consistency with the manuscript.

**Figure 3:**
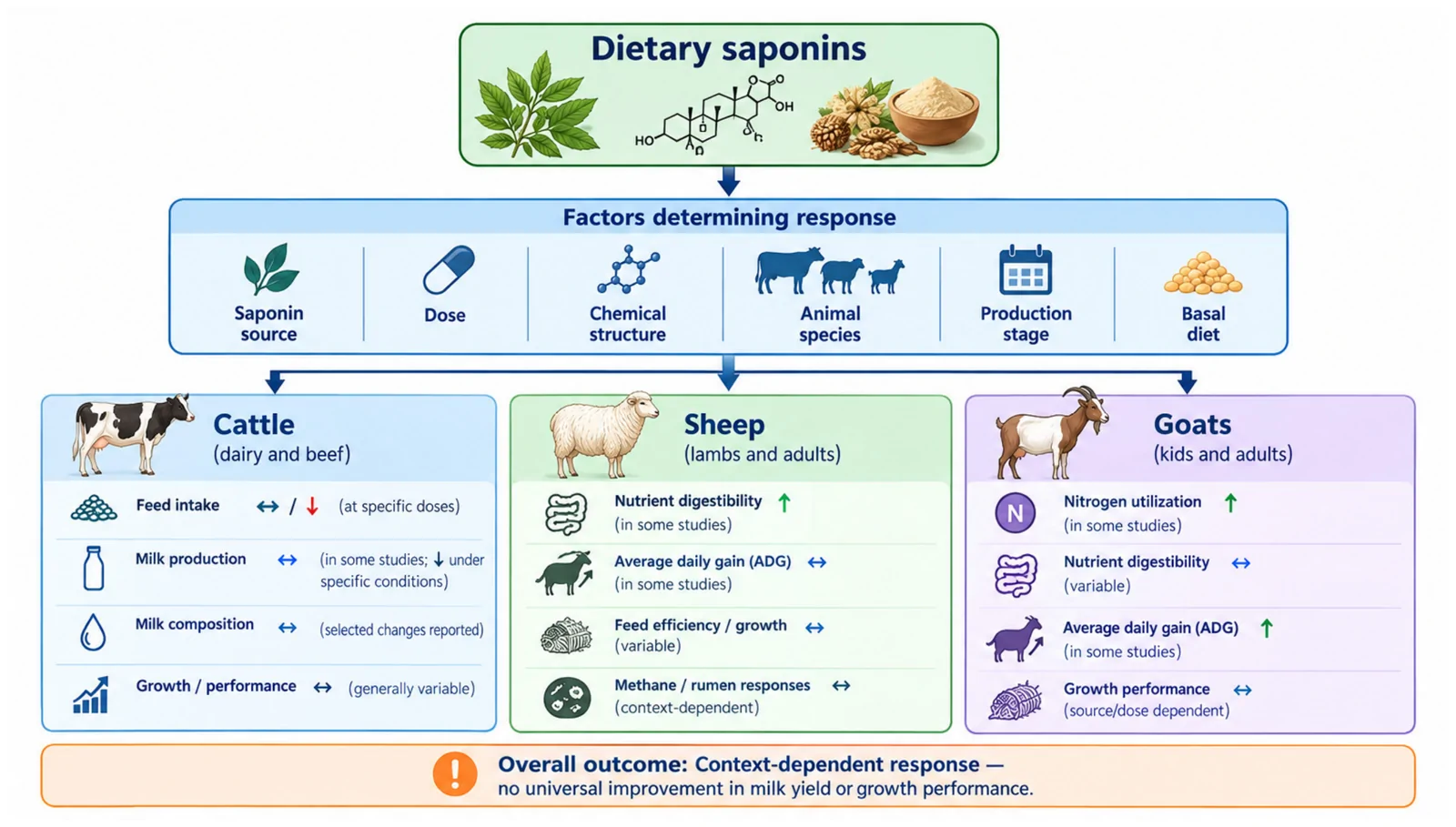
Conceptual summary of the context-dependent effects of dietary saponins on nutrient utilization and productive performance in ruminants. Saponin supplementation may influence feed intake, nutrient digestibility, nitrogen utilization, milk production, and growth performance; however, the direction and magnitude of these responses vary according to saponin source, chemical structure, dose, animal species, production stage, and basal diet. In dairy cattle, milk production may remain unchanged under some supplementation conditions, whereas reductions have been reported under specific doses or experimental conditions. In sheep and goats, improvements in nutrient utilization or growth performance have been reported in some studies, while other studies have found no significant effects. Thus, the pathways and outcomes shown represent potential and study-dependent responses rather than universal effects of dietary saponins. The figure was prepared with assistance from the web-based version of Google NotebookLM and was subsequently reviewed and verified by the authors for scientific accuracy and consistency with the manuscript.

**Figure 4:**
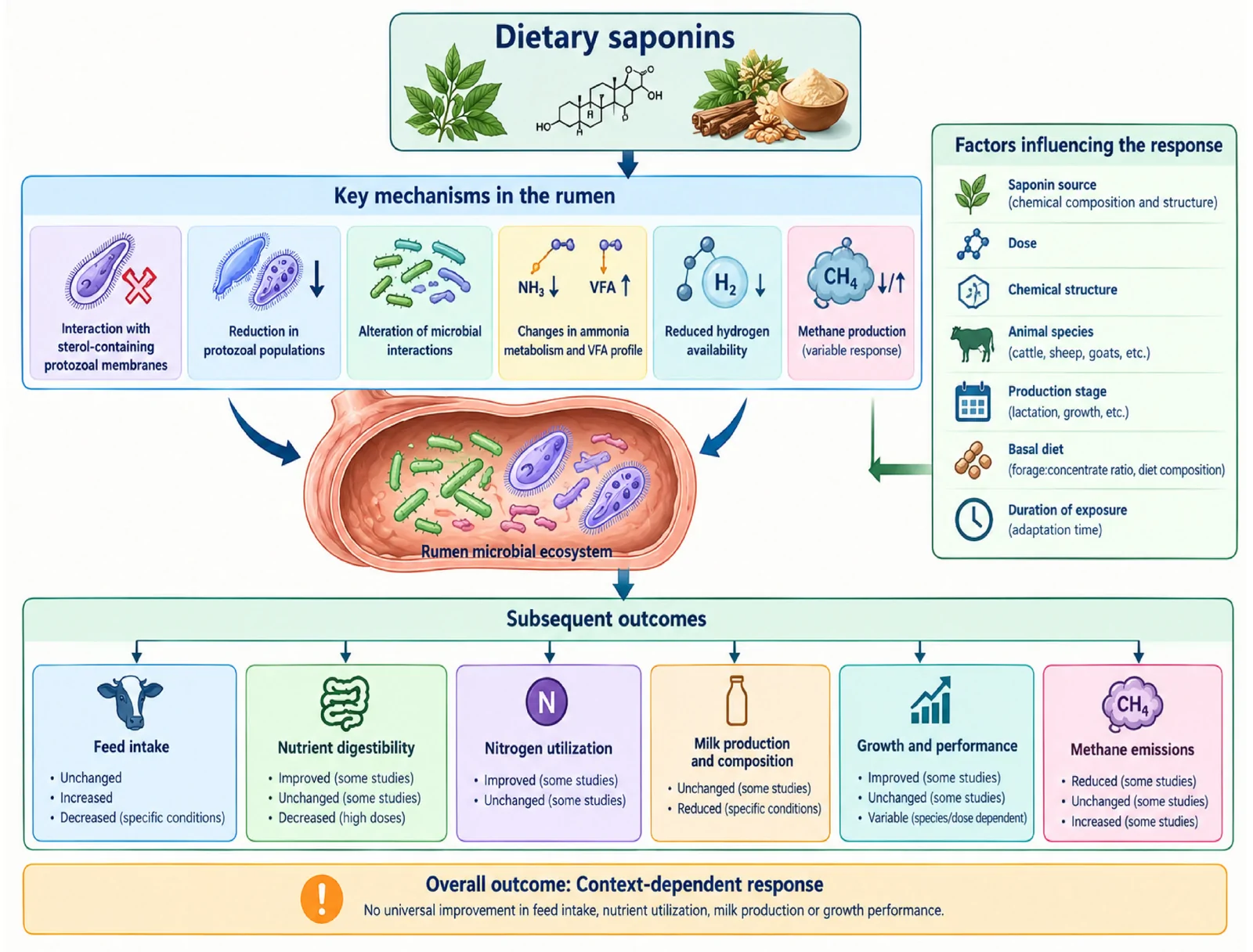
Systemic effects of dietary saponins on metabolic, antioxidant, immune, and endocrine parameters in ruminants. Saponins may reduce blood glucose and triglycerides, lower blood urea nitrogen, enhance antioxidant enzyme activity (SOD, catalase), and modulate immune markers. Endocrine effects are generally limited at moderate doses. Duplicate icons have been removed, and all labels are legible. Abbreviations: SOD, superoxide dismutase; MDA, malondialdehyde; BUN, blood urea nitrogen; IGF-1, insulin-like growth factor 1. The figure was prepared with assistance from the web-based version of Google NotebookLM and was subsequently reviewed and verified by the authors for scientific accuracy and consistency with the manuscript.

Figure 2, Figure 3 and Figure 4 were prepared using the web-based version of Google NotebookLM (Google LLC, USA). The figures were generated based on the scientific information and references reviewed and cited in this manuscript. Following generation, all figures were critically evaluated and checked by the authors for scientific accuracy, consistency with the reviewed literature, and alignment with the content of the manuscript. The use of generative AI was limited to assisting with the visualization and graphical presentation of the scientific information, while the scientific content, interpretation, and final approval of the figures remained the responsibility of the authors.

## 3. Classification of Saponins

**Figure 1:**
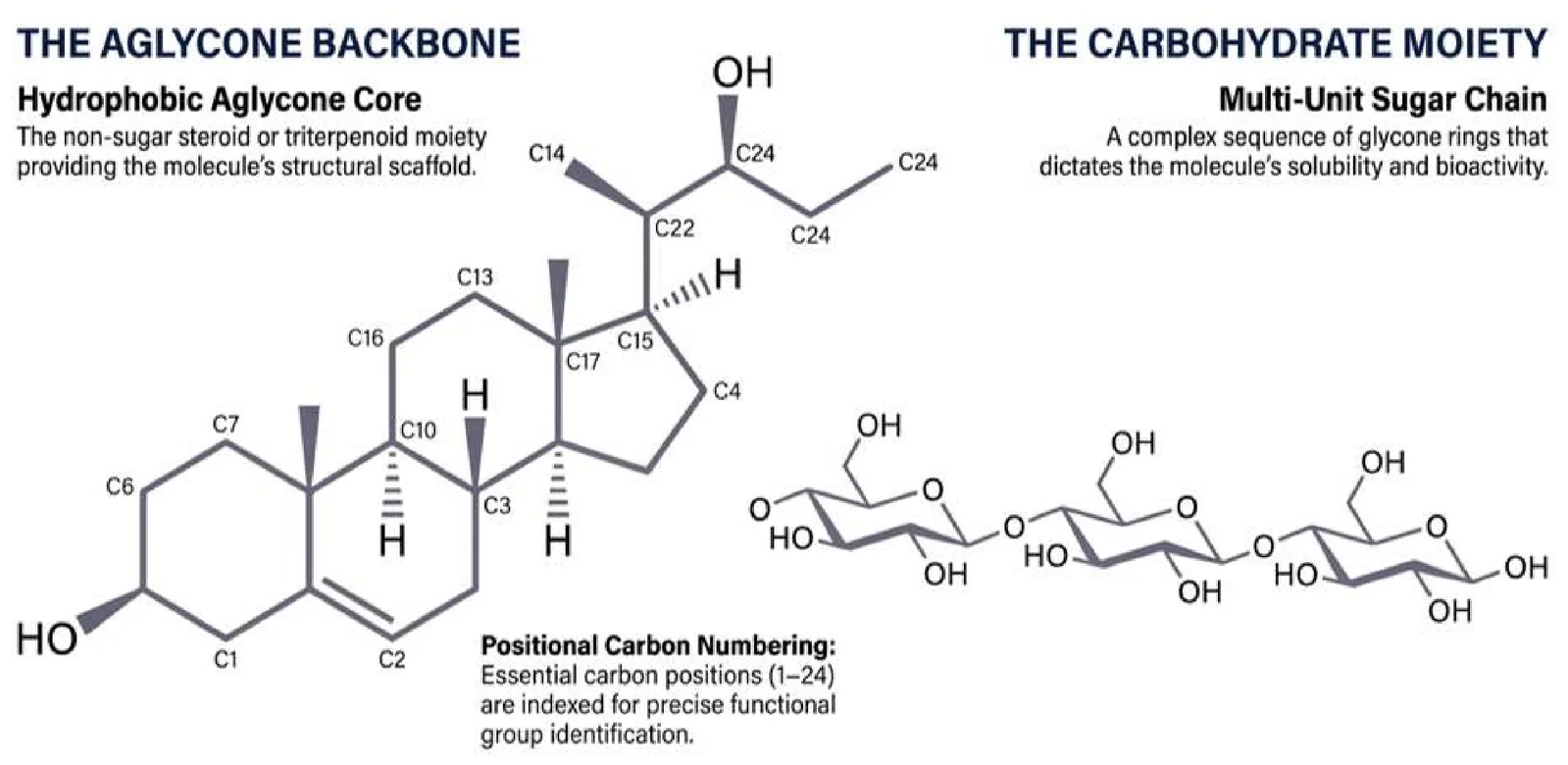
Structure of saponins.

Saponins are naturally occurring amphiphilic glycosides consisting of a hydrophobic aglycone (sapogenin) linked to hydrophilic sugar chains via glycosidic bonds [14,15,16]. Based on the aglycone structure, saponins are classified into two major groups: steroidal (C27) and triterpenoid (C30) saponins. Triterpenoid saponins are further subdivided into tetracyclic and pentacyclic types (Table 1, Figure 1) [17].

Steroidal saponins include spirostanol, isospirostanol, furostanol, steroidal alkaloids, and cholestane derivatives [18,19]. Plant sources such as *Yucca schidigera*, *Agave* spp., and *Dioscorea* spp. are rich in steroidal saponins, which exhibit antiprotozoal, ammonia-reducing, and fermentation-modulating activities in the rumen [15,20]. However, excessive doses may compromise microbial membrane integrity due to surfactant properties [20].

Triterpenoid saponins are classified as tetracyclic (dammarane, lanostane, cucurbitane) or pentacyclic (oleanane, ursane, friedelane, lupane) [21,22,23,24,25,26,27]. Common sources include *Quillaja saponaria*, tea seed (*Camellia sinensis*), alfalfa (Medicago sativa), and Sapindus spp. [8,28,29]. Triterpenoid saponins, particularly monodesmosidic types, exhibit strong membrane-disrupting activity against protozoa and methanogens, but may also suppress fibrolytic bacteria at high doses [30,31,32,33].

Structure–Activity Relationship (SAR): The biological potency of saponins is governed by:

Aglycone type: Triterpenoid saponins generally exhibit stronger antiprotozoal and antimethanogenic effects than steroidal types [34].

Glycosylation pattern: Monodesmosidic saponins (single sugar chain) have greater membrane activity but lower selectivity than bidesmosidic forms (two sugar chains) [32].

Sugar moiety composition: Highly glycosylated saponins show reduced membrane permeability and lower antimicrobial potency [30].

The structural diversity of saponins directly influences their interactions with ruminal microorganisms and explains the variability in fermentation, methane mitigation, and animal performance responses reported across studies (see Section 4.8 for detailed mechanistic analysis).

Although Table 1 and Figure 1 provide a structural overview, the functional consequences of saponin classification require explicit discussion in the text. The aglycone type is not merely a taxonomic feature; it influences how saponins interact with microbial membranes. Steroidal saponins, such as those from *Yucca schidigera* and *Agave* spp., generally have lower membrane-disrupting activity than triterpenoid saponins but show greater selectivity toward protozoa [35,36,37]. This may explain why *Yucca* extracts often reduce ammonia-N and protozoal numbers without consistently lowering methane. In contrast, pentacyclic triterpenoid saponins, particularly monodesmosidic forms from *Quillaja saponaria* and *Sapindus saponaria*, disrupt sterol-containing membranes more aggressively, producing stronger antiprotozoal and antimethanogenic effects. However, this potency is accompanied by a higher risk of suppressing fibrolytic bacteria, especially *Ruminococcus flavefaciens* and *Fibrobacter succinogenes*, at high doses. Glycosylation further modifies these effects: monodesmosidic saponins have one sugar chain and greater membrane activity but lower selectivity, whereas bidesmosidic saponins have two sugar chains and are generally less membrane-active [38,39,40,41]. Highly glycosylated saponins may therefore preserve fiber digestion but show weaker methane mitigation. These structure–activity relationships help explain why studies using different saponin sources do not produce uniform rumen responses [42].

## 4. The Effect of Saponin on Ruminant Nutrition

Dietary saponins are promising phytogenic feed additives. They modulate rumen microbial ecology and improve nutrient utilization. Their amphiphilic structure enables selective interactions with sterol-containing microbial membranes. These interactions primarily affect ruminal protozoa. Reduced protozoal populations indirectly suppress symbiotic methanogenic archaea. Consequently, ruminal fermentation patterns shift toward lower ammonia nitrogen, improved nitrogen retention, and altered VFA profiles. These changes often include increased propionate production and a reduced acetate-to-propionate ratio. As a consequence, dietary saponins may enhance microbial protein synthesis, improve feed utilization efficiency, and contribute to lower enteric methane emissions, thereby supporting both animal productivity and environmental sustainability. Nevertheless, the magnitude and consistency of these responses remain highly variable and are influenced by the botanical origin of the saponin, aglycone structure, glycosylation pattern, dietary composition, supplementation level, animal species, and the adaptive capacity of the rumen microbiota. Excessive supplementation may impair fibrolytic activity and fiber digestibility, highlighting the importance of optimizing inclusion rates according to dietary and physiological conditions. Overall, current evidence suggests that dietary saponins represent promising functional feed additives for sustainable ruminant production; however, their successful application depends on a better understanding of structure-activity relationships and dose-dependent biological responses [15,43].

Table 2 summarizes source-specific outcomes, but these differences should not be read as equivalent effects. *Yucca schidigera* (steroidal) consistently reduces ammonia-N and often decreases protozoa, yet its methane response is variable and sometimes absent, likely because steroidal saponins are less membrane-disruptive and because rumen microbes adapt.

*Quillaja saponaria* (triterpenoid, pentacyclic) shows more consistent methane reduction, averaging 15–21% in some analyses, but high doses can reduce fiber digestibility. Tea seed saponins (*Camellia sinensis*) illustrate a species-dependent paradox: they reduce methane and protozoa in sheep, but in dairy cows they may increase methane per kg DMI or have no effect, possibly due to faster passage rate, lower rumen retention time, and microbial adaptation. Alfalfa saponins (medicagenic acid) mainly improve nitrogen utilization, with little or no methane effect. *Sapindus saponaria*, rich in monodesmosidic saponins, has strong in vitro antimethanogenic activity, but in vivo evidence is limited [48,49,50,51]. Fenugreek steroidal saponins have little effect on methane but may increase milk fat. Mixed saponin–tannin formulations often produce more consistent methane reductions, but responses vary with dose and diet. Thus, the table shows that botanical source, aglycone class, and glycosylation pattern create distinct biological profiles rather than a single common response [55,56,57].

### 4.1. Effects on Feed Intake and Nutrient Utilization

Dietary saponins influence feed intake and nutrient utilization mainly via rumen microbial interactions, often secondary to antiprotozoal effects (Section 4.2). In lactating dairy cows and growing lambs, moderate supplementation (≤40 g/kg DM) with purified or characterized saponins from Yucca schidigera or Quillaja saponaria generally does not alter dry matter intake (DMI), provided the diet is palatable [58]. However, in sheep and goats, crude extracts rich in monodesmosidic triterpenoid saponins (e.g., from Sapindus saponaria) may reduce DMI above 50–60 g/kg DM, likely due to reduced palatability rather than metabolic suppression [30,32,47]. In beef cattle, tolerance appears higher, and intakes up to 60 g/kg DM have been reported without adverse effects [58].

Digestibility responses depend on saponin structure and diet composition. In growing lambs fed low-quality forage, crude protein digestibility often improves with alfalfa saponins (medicagenic acid, characterized extract) or tea seed saponins (characterized extract), because saponins reduce ruminal deamination and enhance microbial protein synthesis [53,55,59]. Fiber digestibility (NDF, ADF) is the most dose- and source-dependent: at ≤40 g/kg DM, most saponin sources are safe in sheep and cattle, but higher doses (>50 g/kg DM) of monodesmosidic triterpenoid saponins (purified or characterized extracts from *Sapindus* or *Quillaja*) suppress fibrolytic bacteria (*Ruminococcus flavefaciens*, *Fibrobacter succinogenes*), reducing NDF/ADF digestion in both sheep and dairy cows [30,32,60,61]. Organic matter and DM digestibility mirror fiber responses; high doses linearly decrease apparent digestibility in lactating cows and growing lambs [60,61]. In summary, saponins consistently improve nitrogen utilization rather than energy digestibility. Fiber digestion is preserved at moderate doses but compromised at high levels. Optimizing saponin type and dose by diet and species is critical (Table 3) [34,58].

Table 3 compiles dose-dependent responses, but the text should explain the underlying trade-offs. At low to moderate inclusion levels (≤30 g/kg DM), feed intake is usually unchanged, while crude protein digestibility may improve modestly and NDF/ADF digestibility is largely preserved [34,53,55,58]. These responses may be related to the reduction in protozoal populations and ruminal ammonia-N without complete suppression of fibrolytic bacteria [51,61]. As the dose increases above 50 g/kg DM, particularly with monodesmosidic triterpenoid saponins, fibrolytic bacteria may be suppressed, leading to reduced NDF/ADF and organic matter digestibility [30,32,60,61]. Methane reduction may be greater at higher doses; however, this potential benefit may be offset by reduced fiber digestion and, in some cases, reduced milk production [29,55,58,60,61]. The acetate-to-propionate ratio typically decreases as hydrogen is redirected toward propionate, although total VFA concentrations may remain unchanged or vary depending on saponin source, diet, and supplementation conditions [46,51]. Therefore, the optimal dose is not universal and depends on saponin source, dietary forage-to-concentrate ratio, animal species, and experimental conditions [34,58]. This may explain why a dose that produces beneficial responses in sheep can be excessive or less effective in dairy cows [48,49,50,62].

**Table 3:** Dose-Dependent Effects of Dietary Saponins on Nutrient Utilization, Fermentation, and Production.

Response Variable	Low-to-Moderate Dose (≤40 g/kg DM)	High Dose (>50 g/kg DM)	Mechanism	Species/Context Sensitivity	Key References
DMI	- (no change)	↓ (palatability issue)	Reduced palatability; not metabolic	Cattle > sheep/goats	[49,59,63]
CP Digestibility	↑ (modest improvement)	-/slight ↓	Reduced deamination; enhanced MCP synthesis	More pronounced on low-quality forages	[46,51,53,61]
NDF/ADF Digestibility	↔ (preserved)	↓ (suppressed)	Suppression of fibrolytic bacteria (*R. flavefaciens*, *F. succinogenes*)	Monodesmosidic > bidesmosidic	[28,53,61]
OM/DM Digestibility	-	↓ (linear decrease)	Mirror of fiber digestibility	Source-dependent	[53,61]
NH_3_-N	↓ (consistent)	↓↓	Reduced proteolysis and deamination	Strongest evidence across species	[46,51,61]
Total VFA	-/slight ↑	-/variable	Microbial activity shifts	Source-dependent	[46,51,61,64]
Propionate	↑	↑ (plateaus)	Hydrogen redirected from methanogenesis	More pronounced in sheep	[46,51,60]
Acetate:Propionate Ratio	↓	↓↓	Fermentation shift toward propionate	Consistent across studies	[46,51,60]
Methane Production	↓ 15–21% (avg.)	↓ up to 40%	Reduced hydrogen transfer to methanogenic archaea	Highly variable; adaptation attenuates over time	[29,49,50,51,58]
Milk Yield	-	↓ at high doses	Reduced DMI; metabolic shift	Dairy cows > beef cattle	[49,56,63]
ADG	-/slight ↑	-	Improved N utilization not always translated to growth	Species-specific	[45,53,59,65,66]
Antioxidant Status	↑ (moderate)	-/variable	Reduced oxidative stress markers (MDA, ROS); increased SOD, catalase	More evident in stressed animals	[63,66]
Inflammatory Markers	↓ (moderate)	-/variable	Cytokine modulation; reduced leukocyte counts	Limited evidence; mechanism unclear	[66,67]

Abbreviations: DM = dry matter; NDF = neutral detergent fiber; ADF = acid detergent fiber; MCP = microbial protein; VFA = volatile fatty acids; ADG = average daily gain; MDA = malondialdehyde; SOD = superoxide dismutase; ROS = reactive oxygen species. ↓ = decrease; ↓↓ = marked decrease; ↑ = increase; - = no significant effect; ↔ = no clear change or a comparable response relative to the control or baseline value.

### 4.2. Effects on Rumen Fermentation and Methane Production

In sheep and goats, purified or characterized triterpenoid saponins from tea seed (*Camellia sinensis*) and *Quillaja saponaria* act primarily by interacting with sterol-containing protozoal membranes, reducing protozoa (defaunation) [15,43,51]. This indirectly suppresses methanogenic archaea that live symbiotically with protozoa, reducing hydrogen transfer to methanogenesis [15,43,51]. However, in lactating dairy cows, this effect attenuates over time due to microbial adaptation (deglycosylation, membrane remodeling; Section 5.2), and tea saponins may even increase methane per kg DMI [48,49,50,62].

Ammonia-N reduction: Lower protozoal predation increases bacterial protein turnover and microbial protein outflow to the duodenum; decreased proteolysis and deamination lower ruminal NH_3_-N, a consistent indicator of improved nitrogen efficiency, supported by increased urinary purine derivatives [51,61].

VFA shifts: Defaunation typically shifts VFA production toward propionate (and sometimes butyrate), decreasing the acetate:propionate ratio [46,51]. This is energetically favorable as propionate production consumes hydrogen otherwise used for methanogenesis. The magnitude varies with saponin source, diet forage:concentrate ratio, and supplementation duration; total VFA may remain unchanged or slightly increase [46,51].

Methane emissions: The most sought-after but most inconsistent effect. Meta-analyses report average reductions of 15–21% in methane production and 15% in methane yield across ruminant species [29,58]. However, individual studies range from 40% inhibition to no effect or even increases (e.g., tea saponins in lactating dairy cows [49]). These contradictions arise from differences in saponin structure, dose, species (sheep vs. cattle), diet, microbial adaptation, and the nature of the test material (purified saponins vs. crude extracts vs. mixtures) (Section 4.8 and Section 5.2) [49,50,62]. In conclusion, saponins consistently reduce NH_3_-N and alter VFA profiles in sheep, goats, and cattle, but methane responses are highly context-dependent. Successful mitigation requires careful source selection, dose optimization (typically ≤40 g/kg DM for sustained efficacy without compromising fiber digestion), and adaptation management [34,58].

### 4.3. In Vivo and In Vitro Responses

In vivo responses to dietary saponins vary considerably according to saponin source, animal species, production stage, dietary conditions, and duration of supplementation [49,56]. Among lactating dairy cows, contrasting responses have been reported for different saponin sources. Supplementation with 0.52% tea seed saponins (Camellia sinensis, triterpenoid) did not reduce daily methane production; instead, methane production per kg DMI increased by 14%, while gastrointestinal digestibility, nitrogen balance, and protozoal populations remained unchanged [49]. In contrast, supplementation with a saponin–tannin blend containing saponins from Yucca schidigera and quebracho tannins reduced enteric CH4, CO2, and N2O emissions without compromising production, although manure ammonia emissions increased [56]. Thus, these contrasting responses occurred under the same broad production category (lactating dairy cows) and are more appropriately interpreted in relation to differences in saponin source and formulation rather than animal type.

In vitro studies provide additional evidence that the response to saponins is strongly dependent on source and inclusion level. Saponin-containing plants, including Yucca schidigera and Quillaja saponaria, have been reported to decrease methane production and ruminal ammonia-N and to lower the acetate-to-propionate ratio, although these effects may be accompanied by reductions in DM and NDF digestibility at higher concentrations [28]. At a lower supplementation level (10 g/kg DM), no significant effects on methane production, fermentation characteristics, digestibility, or milk yield were observed in dairy cows [28]. Similarly, an herbal formulation containing 30% saponins produced a quadratic response in CP digestibility in growing lambs [60]. These findings indicate that the magnitude and direction of the response depend not only on the presence of saponins but also on their concentration and the characteristics of the test material.

Species-related differences become more apparent when in vivo studies in small and large ruminants are considered. In growing lambs, saponin supplementation reduced ruminal NH3-N, increased total VFA concentration, and decreased methane emissions, with a concomitant reduction in the acetate-to-propionate ratio [46]. Tea saponins also slightly decreased ruminal pH, increased total VFA and microbial protein synthesis in growing lambs [64], while higher saponin levels reduced NH3-N and protozoal populations and improved nitrogen efficiency in goats [61]. However, increasing saponin levels in goats also linearly decreased the apparent digestibility of DM, OM, CP, and NDF, despite improvements in microbial protein synthesis [61]. In Hu sheep, tea saponins reduced methane production and protozoal abundance and increased propionate production without adversely affecting fibrolytic microbes [51]. In contrast, responses in cattle have generally been less consistent. Tea seed saponins did not reduce methane production in cattle, and cessation of supplementation was associated with an 18–22% decrease in daily methane production [50]. Tea saponins also increased protozoal populations during supplementation, which returned to baseline after withdrawal [50]. Other studies in dairy cows reported no significant effects of tea saponins on small-intestinal amino acid digestibility or rumen fermentation [52]. In addition, supplementation with 3 g/day of Yucca schidigera extract did not significantly affect protozoal populations, ruminal fermentation, digestibility, or milk yield in dairy cows [62].

Overall, the available evidence indicates that differences among studies cannot be attributed to animal species alone. Rather, species and production stage interact with saponin source, chemical structure, supplementation level, basal diet, and duration of exposure. Responses appear to be more consistently favorable for methane mitigation and nitrogen utilization in several sheep and goat studies, whereas responses in cattle, particularly lactating dairy cows, are more variable [49,50,51,61,62]. These differences may reflect variation in saponin degradation, ruminal retention time, microbial adaptation, dietary conditions, and experimental design. Therefore, comparisons among studies should consider animal characteristics together with the properties of the saponin preparation and the feeding conditions rather than treating species or production stage as independent explanatory factors [49,50,62,68].

### 4.4. Milk Yield, Milk Quality, Feed Intake, and Growth Performance

The effects of dietary saponins on productive performance in ruminants are variable and appear to depend on saponin source, supplementation level, animal species, and dietary conditions. In lactating dairy cows, a saponin–tannin blend did not significantly affect dry matter intake (DMI), energy-corrected milk (ECM) production, or milk fat and protein yields compared with the control group [56]. Similarly, supplementation with Micro-Aid^®^, a product containing Yucca schidigera saponins, did not significantly affect forage intake or milk production in beef cows [45]. However, responses were not consistently neutral. In another study with lactating dairy cows, supplementation with a saponin–tannin combination resulted in an 18% decrease in milk production, accompanied by a 12% reduction in DMI and an 8% decline in feed efficiency [49]. Administration of 2 g/day of tea saponins to dairy cows also reduced DMI and resulted in the lowest yields of milk, milk protein, fat, and lactose [63]. These findings suggest that sufficiently high or specific combinations of saponins may adversely affect milk production, particularly when reductions in feed intake occur.

Regarding milk composition and quality, the available evidence indicates that saponins can modify selected milk components without necessarily altering total milk production. Supplementation with 60 g/day of *Yucca schidigera* saponin extract caused minor changes in the milk fatty acid profile of dairy cows [69]. The proportions of caproic acid (C6:0), caprylic acid (C8:0), and vaccenic acid (trans-11 C18:1) decreased from 2.95% to 2.71%, 1.89% to 1.66%, and 1.01% to 0.92%, respectively [63]. However, conjugated linoleic acid (CLA; cis-9, trans-11 C18:2), linoleic acid, and α-linolenic acid were not significantly affected [69]. In another study, milk fat content increased linearly with increasing tea saponin supplementation, although intermediate doses of 1 and 1.5 g/day did not significantly affect feed intake [63]. Furthermore, supplementation with fenugreek-derived saponins alone or in combination with essential oils did not alter overall milk yield or nutrient digestibility but increased milk fat and protein concentrations, suggesting that changes in milk composition may occur independently of changes in total milk production [57].

The effects of saponins on feed intake are likewise inconsistent across studies. Several investigations reported no significant changes in DMI following saponin supplementation. Dietary tea saponins, for example, did not significantly affect feed intake, nutrient digestibility, or nutrient disappearance from the forestomach of dairy cows; apparent digestibility of DM, nitrogen, NDF, and ADF also remained unchanged [52]. Similarly, saponin supplementation did not significantly affect feed intake, final body weight, or average daily gain in growing lambs [64]. The inclusion of saponins and tannins from leguminous plants also did not adversely affect DMI in heifers [60]. Administration of Agave americana or Quillaja saponaria extracts to Barbarine female lambs did not significantly influence feed intake, nutrient digestibility, nitrogen balance, or microbial protein synthesis [65].

In contrast, some studies reported increased feed intake following supplementation. Aregheore found that *Yucca schidigera* saponins increased DMI, daily weight gain, digestible protein intake, metabolizable energy intake, and apparent digestibility of DM, CP, organic matter, and energy in goats [64]. Similarly, sheep receiving higher doses of saponin-containing plants showed increased intake of DM, CP, crude fiber, and energy-yielding nutrients, together with improved nutrient utilization, feed efficiency, and growth performance [67]. These findings indicate that saponins do not exert a uniform effect on feed intake; rather, their effects may depend on source, dose, palatability, animal species, and the composition of the basal diet.

With respect to growth performance, responses are also heterogeneous. In growing lambs, supplementation with alfalfa-derived saponins for 90 days linearly improved the digestibility of DM, CP, and ADF but did not significantly affect average daily gain, feed conversion ratio, body length, or chest circumference [53]. Likewise, supplementation with Agave americana or Quillaja saponaria extracts produced a numerical increase in ADG in Barbarine lambs, although the differences were not consistently statistically significant [65]. Conversely, lambs receiving a combined treatment of yucca extract and organic chromium exhibited greater body weight gain and ADG than control animals and those receiving either supplement alone [66]. McMurphy et al. also reported greater body weight in calves nursing supplemented beef cows at the end of the supplementation period, although this difference was not maintained until weaning [45].

Overall, the evidence indicates that the effects of dietary saponins on milk production, milk composition, feed intake, and growth performance are highly context-dependent. Neutral responses are common, whereas positive or negative effects have generally been associated with supplementation level, saponin source and structure, animal species, basal diet, and duration of exposure [34,58]. Thus, changes in productive performance should be interpreted together with changes in feed intake and nutrient utilization rather than considered as isolated responses (Figure 3).

### 4.5. Effects of Saponins on Reproductive Function in Ruminants

Reproductive efficiency is key to sustainable ruminant production, and emerging evidence suggests dietary saponins may influence reproduction through indirect metabolic pathways rather than direct endocrine effects, though this area remains less consistent than rumen fermentation research [70]. In dairy cows and heifers, the primary mechanism appears indirect: by reducing ruminal ammonia and enhancing microbial protein synthesis, saponins improve overall metabolic status, particularly during the transition from late gestation to early lactation when negative energy balance compromises ovarian function [23]. Additionally, their antioxidant properties may protect ovarian tissues from oxidative stress, which impairs oocyte quality, embryo survival, and luteal function [70]. Saponin-rich plants like fenugreek and shatavari have been suggested to support reproduction in sheep and goats, partly through phytoestrogen-like activities [12]. Some positive associations have been reported: *Yucca schidigera* extract improved reproductive efficiency in heifers and dairy cows, attributed to reduced blood ammonia and better metabolic health [12]. Tea saponins improved body weight and serum parameters in lambs, potentially benefiting future reproduction [12]. However, direct endocrine effects remain inconclusive. Triterpenoid saponins from *Saponaria officinalis* altered blood metabolites but did not consistently affect reproductive hormones (insulin, glucagon, thyroid hormones) in sheep, suggesting metabolic rather than HPG axis modulation [71]. Though steroidal saponins resemble cholesterol and could interfere with steroidogenesis, this has not been confirmed in vivo and may be dose-dependent [30]. Inconsistencies arise from saponin type, source, dosage, physiological state, and lack of studies designed for reproductive endpoints [30]. Meta-analyses show species-specific responses: cattle differ from sheep and goats [34,58]. While up to 40 g/kg DM appears safe for intake, higher doses may pose reproductive risks [30]. In conclusion, reproductive benefits are largely indirect, via improved metabolism, nitrogen utilization, and antioxidant defense—rather than direct endocrine regulation [34,72]. Saponin-rich plants show promise, but well-controlled long-term studies measuring conception rates, calving intervals, and ovarian function are urgently needed in cattle, sheep, and goats [34,72]. Future research should explore synergistic combinations with tannins or essential oils to optimize metabolic health and fertility [34,72].

### 4.6. Blood Metabolites, Antioxidant Status, and Immunological Indices

Beyond their effects on rumen fermentation and nutrient utilization, dietary saponins have been shown to influence systemic metabolic parameters in ruminants, reflecting their absorption and bioactivity beyond the gastrointestinal tract [44,55]. These systemic effects are of particular interest because they may contribute to overall animal health and resilience to disease [44].

Glucose, lipid, and nitrogen metabolites: Several studies in growing lambs and dairy cows have reported reductions in blood glucose and triglyceride concentrations following saponin supplementation, suggesting modulation of carbohydrate and lipid metabolism [53,65]. Liu et al. observed a significant decrease in plasma glucose and triglycerides in lambs fed increasing doses of alfalfa saponins [53], while Nasri et al. reported similar cholesterol-lowering effects in lambs receiving *Quillaja saponaria* extract [65]. In dairy cows, supplementation with saponins alone or in combination with essential oils reduced blood urea nitrogen (BUN), indicative of improved nitrogen utilization efficiency [57]. These metabolic improvements are likely mediated through enhanced ruminal nitrogen capture and altered hepatic metabolism rather than through direct hormonal regulation [57].

Antioxidant status: The antioxidant properties of saponins have been demonstrated in several ruminant studies [63,66]. Wang et al. reported that tea saponin supplementation in dairy cows decreased plasma malondialdehyde (MDA) concentrations and increased superoxide dismutase (SOD) activity, suggesting mitigation of oxidative stress [63]. Similarly, Griss et al. found that a combination of yucca extract and organic chromium enhanced antioxidant enzyme activities (catalase, SOD) while reducing reactive oxygen species in lambs [66]. These effects may have important implications for reproductive function (see Section 4.5) and immune competence [63,66].

Inflammatory and immune markers: Saponins have demonstrated immunomodulatory properties in both in vitro and in vivo studies [66,67]. In lambs supplemented with yucca extract and chromium, serum total protein and globulin concentrations increased, while leukocyte, neutrophil, and lymphocyte counts decreased, suggesting an anti-inflammatory effect without immunosuppression [66]. Similarly, in parasitized sheep, saponin-rich anthelmintic treatments maintained hematological parameters within physiological norms while improving overall health status [67]. These findings align with the broader literature on saponin bioactivity in ruminants, though the exact mechanisms remain poorly defined [34].

Endocrine parameters: Evidence for direct effects on metabolic hormones is limited and inconsistent [53,55]. Liu et al. found no significant changes in growth hormone, insulin-like growth factor 1 (IGF-1), or thyroid hormones in lambs fed alfalfa saponins, suggesting that saponins do not directly disrupt the endocrine axis at nutritionally relevant doses [53]. In summary, dietary saponins appear to exert beneficial systemic effects on metabolic status, antioxidant defense, and immune function in cattle, sheep, and goats, likely as a consequence of improved rumen nitrogen metabolism and direct bioactive properties [34,58]. These effects are generally subtle and dose-dependent, and while they do not dramatically alter endocrine profiles at moderate doses, they may contribute to improved animal health and resilience, especially under conditions of metabolic or oxidative stress (Figure 4) [34,58].

### 4.7. Meat Quality

Meat derived from lambs treated with saponins demonstrated reduced lipid oxidation relative to that from the tannin-treated group, a phenomenon likely attributable to the antioxidant properties inherent in the saponin extract [73]. Additionally, the concentration of lactate (2-hydroxypropanoic acid) was found to be lower in the muscle tissue of the saponin-treated group, indicating a potential inhibitory effect of saponins on rumen protozoa, which may subsequently promote an increase in lactate-utilizing bacterial populations [73]. Furthermore, saponin administration resulted in a decrease in cholesterol levels within the meat, corroborating previous research that suggests saponins possess an anti-cholesterolemic effect [73]. Importantly, parasite control treatments incorporating saponins do not appear to adversely impact the quality of sheep meat [73]. In a related study, Nasri et al. reported that dietary supplementation with *Quillaja saponaria* extract enhanced the color stability of lamb meat during refrigerated storage without negatively affecting lipid oxidation or the composition of volatile compounds [65,74]. These findings imply that *Quillaja saponaria* extract may function as a natural feed additive to improve meat quality and extend shelf life in sheep [65,74]. However, further research is necessary to delineate the specific contributions of saponins as distinct from other bioactive constituents present in the extract [74]. Although limited information is available regarding the effects of saponins on meat quality in cattle or goats, current evidence suggests beneficial impacts on oxidative stability, cholesterol reduction, and color preservation without adverse effects on overall meat quality characteristics (Table 2 and Table 4) [34,73,74].

### 4.8. Deconstructing Inconsistencies: Mechanistic Drivers of Variable Responses

A critical examination of the literature reveals that the effects of dietary saponins on rumen fermentation, methane emissions, and animal performance are far from uniform across species, production stages, saponin sources, and test materials [34,49,50,62]. While some studies using purified or characterized saponins in sheep report substantial methanogenesis inhibition (up to 40%) and improved nitrogen utilization, others using crude extracts or mixtures in dairy cows demonstrate no effect or even adverse responses [49,50,51,62,68]. These contradictions are not random; they originate from identifiable mechanistic drivers that can be categorized into four interrelated levels: (i) saponin structural chemistry, (ii) rumen microbial ecology and adaptation, (iii) host physiological factors, and (iv) dietary matrix interactions [30,32,34,49,50,60,62].

#### 4.8.1. Structural Chemistry as a Primary Determinant

The degree of glycosylation and the configuration of the aglycone profoundly influence biological potency [30,32]. Monodesmosidic saponins (single sugar chain), such as those predominant in *Sapindus saponaria*, exhibit greater membrane-disrupting activity than bidesmosidic forms (two sugar chains), which are more common in alfalfa and soybean [30,32]. However, this higher potency comes at the cost of reduced selectivity; monodesmosidic saponins can suppress fibrolytic bacteria (e.g., *Ruminococcus flavefaciens*) alongside protozoa, thereby impairing NDF digestibility at high doses in both sheep and cattle [30,32]. Conversely, highly glycosylated triterpenoid saponins, such as those from tea seed (*Camellia sinensis*), show weaker antimicrobial activity but better preservation of fiber-digesting populations, explaining why tea saponins often reduce methane in sheep but fail to do so in dairy cattle [49,51]. This species-specific discrepancy is further magnified by differences in rumen retention time and protozoal community composition between small and large ruminants [49,51].

#### 4.8.2. Microbial Adaptation: The Hidden Attenuator

Perhaps the most underappreciated driver of inconsistency is the adaptive capacity of rumen microbiota [46,50,62]. Prolonged saponin supplementation frequently leads to diminished antiprotozoal effects over time, as certain protozoal species develop resistance through altered membrane sterol composition, and bacterial populations capable of deglycosylating saponins into less toxic sapogenins proliferate [46,50,62]. This adaptation explains why short-term in vitro studies typically report stronger antimethanogenic effects than long-term in vivo trials in cattle and sheep [49,50]. For instance, the disappearance of the methane-suppressive effect in dairy cows fed tea saponins for several weeks contrasts sharply with the sustained responses observed in short-term sheep experiments [49,51]. This temporal attenuation suggests that efficacy depends not only on the saponin itself but also on the duration of exposure and the pre-existing microbial baseline [49,50,62].

#### 4.8.3. Host Physiology and Dietary Matrix Effects

Ruminant species differ substantially in rumen volume, passage rate, and protozoal diversity [34,49,51]. Dairy cows, with higher feed intake and faster passage rates, may experience reduced saponin-microbe contact time compared to sheep or goats, partially explaining why bovine responses are often weaker [34,49]. Additionally, the dietary forage-to-concentrate ratio modulates rumen pH and the bioavailability of saponins; high-grain diets lower pH, potentially altering the membrane-interactive efficiency of saponins, while high-forage diets promote fibrolytic communities that are more susceptible to inhibition [60]. The presence of dietary tannins, which can form complexes with saponins, further complicates predictions by either synergistically enhancing antiprotozoal effects or antagonistically reducing saponin solubility and bioavailability [56,57,60].

#### 4.8.4. Nonlinear Dose–Response and the Absence of a Demonstrated Hormetic Effect

The relationship between saponin dosage and biological response is frequently nonlinear [58]. Beneficial effects on nitrogen utilization and propionate production typically plateau at moderate inclusions (approximately 20–40 g/kg DM), whereas fiber digestibility and DMI decline only when thresholds exceed 50 g/kg DM, depending on the source [58]. This pattern is best described as a biphasic dose–response or nonlinear dose–response, rather than hormesis. A true hormetic response would require evidence of low-dose stimulation followed by high-dose inhibition; such evidence has not been demonstrated for saponins in ruminants. The observed pattern indicates that “more is not better” and that optimal doses are source-specific and diet-dependent, a nuance often overlooked in studies that test only a single concentration [58]. In summary, the apparent contradictions in the saponin literature are largely reconcilable when viewed through the lens of saponin chemistry, microbial adaptation, host physiology, and dietary context [34,58]. Future studies must move beyond simple efficacy testing and adopt factorial designs that systematically vary these factors, combined with metagenomic and metabolomic profiling, to disentangle their interactive effects and enable evidence-based precision supplementation (Table 4) [34,58].

Table 4 lists mechanistic drivers of inconsistency, but these factors interact rather than act independently. Structural chemistry sets the initial potency; monodesmosidic triterpenoids are more membrane-active but less selective. Microbial adaptation then attenuates the effect over weeks, as bacteria deglycosylate saponins and protozoa remodel their membranes. Host physiology modulates exposure: cattle have faster passage rates and larger rumen volumes than sheep, so saponin–microbe contact time is shorter. Dietary context, especially forage-to-concentrate ratio and tannin content, alters pH, solubility, and binding. Finally, dose–response is nonlinear: beneficial effects plateau, while negative effects on fiber digestion appear at high doses. These interactions mean that single-factor studies are unlikely to predict field responses; factorial designs are needed.

#### 4.8.5. Critical Evaluation of Divergent Methane Responses: The Case of Tea Saponins in Dairy Cows

Tea saponins (*Camellia sinensis*) provide a striking example of divergent methane responses. In sheep, tea saponins consistently reduce methane and protozoal populations [51]. In dairy cows, however, 0.52% tea saponin (approximately 5.2 g/kg DM) did not reduce daily methane production; methane per kg DMI increased by 14% [49]. Similarly, in cattle, cessation of tea saponin supplementation led to an 18–22% decrease in daily methane production, implying that supplementation may have increased methane [50]. These contrasting outcomes can be reconciled by considering several confounding factors.

Methane measurement methodology: In sheep studies, methane is often measured by respiration chambers or the SF_6_ tracer technique, which may differ from measurements in dairy cows using GreenFeed or other systems [49,51]. Differences in measurement duration, frequency, and proximity to feeding can affect estimates. For example, GreenFeed systems may underestimate methane emissions if animals do not visit frequently, while respiration chambers provide continuous measurement but may not reflect normal feeding behavior.

Adaptation period: Short-term in vitro studies typically show strong antimethanogenic effects, but long-term in vivo trials often show attenuation. In dairy cows, tea saponins were fed for several weeks, allowing microbial adaptation (deglycosylation, protozoal membrane remodeling) to occur [49,50,62]. In sheep, responses may be sustained longer due to a slower passage rate and a different protozoal community [51].

Diet composition: Dairy cows are typically fed high-grain diets, which lower rumen pH and may alter saponin bioavailability and membrane-interactive efficiency [60]. High-forage diets in sheep may promote fibrolytic communities more susceptible to saponin inhibition. The forage-to-concentrate ratio also affects rumen retention time and microbial community structure, which in turn influence saponin efficacy.

Dose: The dose in dairy cows (0.52% of diet, approximately 5.2 g/kg DM) may be below the threshold for sustained methane inhibition in large ruminants, or may be in a range where adaptation occurs rapidly [49]. In sheep, effective doses are often higher relative to body weight or rumen volume [51]. The nonlinear dose–response relationship (hormesis) means that low doses may fail to inhibit methanogens while high doses may suppress fiber digestion [58].

Baseline protozoal community structure: Dairy cows may have different protozoal populations (e.g., higher *Entodinium* spp.) that are less sensitive to tea saponins or can remodel membrane sterols more effectively [75,76]. In sheep, the protozoal community may be more susceptible. The initial protozoal community structure therefore determines the magnitude and duration of the antiprotozoal effect.

Reconciliation with the SAR framework: The SAR framework predicts that triterpenoid saponins with bidesmosidic structures (such as tea saponins) have lower membrane activity and better preservation of fiber digestion but weaker methane mitigation [30,32]. This is consistent with tea saponins in dairy cows: they fail to reduce methane and may even increase methane per kg DMI due to adaptation and diet effects [49]. In sheep, the same saponin may still reduce methane because of slower passage, a different protozoal community, and possibly a lower deglycosylation rate [51]. Thus, the SAR framework should incorporate host species, diet, and microbial adaptation as moderators of saponin efficacy. Future studies should report methane measurement methodology, adaptation period, diet composition, dose, and baseline protozoal community to allow better cross-study comparison [34,58].

### 4.9. Quantitative Insights from Recent Meta-Analyses: Toward Evidence-Based Generalization

To move from qualitative description to quantitative generalization, several recent meta-analyses have pooled data across studies, providing statistically robust estimates of saponin efficacy. However, these syntheses also reveal substantial heterogeneity that must be interpreted in light of moderator variables.

#### 4.9.1. Specific Meta-Analyses Supporting Methane Reduction Estimates

In vivo meta-analysis [58]: Yanza et al. evaluated 26 papers (66 in vivo studies, 148 comparisons) across ruminant species. Saponin supplementation up to 40 g/kg DM had no detrimental impact on feed intake, establishing a safety threshold. Species-specific responses were observed, with larger ruminants better able to tolerate bitterness. Among saponin sources, Quillaja saponaria extract was more likely to improve large ruminant production performance while maintaining health, but negatively affected small ruminants. Increased supplementation linearly decreased milk production and altered VFA profiles and protozoal populations.

Methane mitigation meta-analysis [77]: Pepeta et al. compiled data from 110 refereed publications on in vivo studies in ruminants from 2005 to 2023. Of nine dietary rumen-modulating strategies evaluated, saponins reduced CH_4_ production on average by 21%, while CH_4_ yield was reduced by 15% on average. In dairy ruminants, saponins reduced CH_4_ emission intensity (g CH_4_/kg milk) by 28.7% on average.

In vitro meta-analysis [78]: Yanza et al. (2026) evaluated 277 in vitro studies. Saponin extracts decreased CH_4_ production by 14.8%, comparable to plant extracts (−14.5%) and tannins (−14.5%), though less potent than chemical inhibitors (−29.2%). Subgroup analysis revealed divergent effects among saponin sources: Calendula officinalis, Knautia arvensis, and tea (Camellia sinensis) increased VFA production, while other sources showed no effect.

Saponin-rich source meta-analysis [29]: Jayanegara et al. [29] conducted a meta-analysis on methane-mitigating properties of saponin-rich sources in the rumen, focusing on the influence of addition levels and plant sources. This analysis confirmed dose-dependent effects and source-specific variation, with saponin-rich sources possessing a comparative advantage over tannin-rich sources in mitigating CH_4_ emission.

#### 4.9.2. Moderators of Saponin Efficacy

The apparent inconsistencies in methane reduction estimates are not random but are systematically influenced by several moderator variables:

Animal species: Responses differ substantially between cattle and small ruminants. In the meta-analysis by Pepeta et al. [77], saponin effects on CH_4_ production were observed across beef cattle, buffaloes, goats, and sheep under both confined and grazing systems. Yanza et al. [58] demonstrated that larger ruminants (cattle) tolerate saponin bitterness better than small ruminants and respond differently in terms of nutrient absorption efficiency. Tea saponins, for example, reduce methane and protozoa in sheep [51] but may increase methane per kg DMI in dairy cows [49].

Diet composition: The forage-to-concentrate ratio modulates rumen pH and saponin bioavailability. High-grain diets lower pH, potentially altering membrane-interactive efficiency, while high-forage diets promote fibrolytic communities more susceptible to inhibition [60]. Basal substrate differences were identified as a key factor governing saponin efficacy in the in vitro meta-analysis [78].

Treatment duration: Adaptation substantially attenuates efficacy over time. The in vitro meta-analysis noted that tea saponin showed absence of methane reduction effect, which may be explained by the requirement for longer adaptation to saponin to exhibit an antiprotozoal effect [78]. This was supported by in vivo findings where 3 weeks of tea saponin supplementation did not alter H_2_ production and CH_4_ [49].

Saponin source and extract type: Different plant sources exhibit distinct effects. In the subgroup meta-analysis by Yanza et al. [78], rumen modulatory effects differed among saponin sources, with tea leaves influencing methane production through two main pathways: direct inhibition of H_2_ production via antiprotozoal activity and VFA modulating effects. The divergent effects reflect fundamental differences in chemical structure and biological activity, including aglycone type (triterpenoid vs. steroidal), number and linkage of sugar moieties, and affinity for membrane sterols [30,32].

#### 4.9.3. Chemical Characterization of Saponin Sources in Cited In Vivo Studies

A critical limitation of the current evidence base is the inconsistent and often incomplete chemical characterization of saponin sources used in in vivo studies. This heterogeneity fundamentally limits cross-study comparability and contributes to apparent contradictions in the literature. The following issues are particularly important:

Total saponin quantification: Most in vivo studies report saponin doses based on total saponin content determined by spectrophotometric methods using diosgenin or other sapogenin standards [30,47]. However, these colorimetric assays do not distinguish between different saponin classes, glycosylation patterns, or biological potencies. Studies using *Yucca schidigera*, *Quillaja saponaria*, tea seed, alfalfa, and *Sapindus* extracts often differ in their standard curves and extraction protocols, making direct dose comparisons problematic [34,47].

Individual sapogenin profiling: Few studies provide quantitative profiles of individual sapogenins. For *Yucca schidigera*, novel methods using gas chromatography-tandem mass spectrometry have been developed to quantify specific sapogenins (e.g., sarsasapogenin, smilagenin, hecogenin) in commercial extracts [44]. However, such detailed characterization is rarely applied in ruminant nutrition studies, where crude extract doses are typically reported without sapogenin-level resolution [34,45].

Mono- vs. bidesmosidic fractions: The ratio of monodesmosidic to bidesmosidic saponins is a critical determinant of biological activity, yet most in vivo studies do not report this ratio [30,32]. Monodesmosidic saponins (single sugar chain) have greater membrane-disrupting activity but lower selectivity, while bidesmosidic forms (two sugar chains) are less membrane-active [30,32]. This distinction explains why *Sapindus* (rich in monodesmosidic saponins) shows strong in vitro antimethanogenic activity but limited in vivo evidence [47,54], whereas alfalfa (rich in bidesmosidic saponins) mainly improves nitrogen utilization with little methane effect [52,53].

Recommendations for future studies: To enable meaningful cross-study comparisons, future in vivo studies should report: (i) total saponin content with the standard used; (ii) aglycone type (steroidal vs. triterpenoid); (iii) mono-/bidesmosidic ratio when determinable; (iv) individual sapogenin profiles for key sources (e.g., sarsasapogenin for *Yucca*, quillaic acid for *Quillaja*); and (v) extraction method and plant part used [34,47]. Standardized commercial extracts with certified saponin content would greatly facilitate this effort.

#### 4.9.4. Synthesis and Implications

Meta-analyses confirm: (1) saponins reduce enteric methane by 15–21% on average, with Pepeta et al. [77] reporting 21% reduction in CH_4_ production and 15% in CH_4_ yield; (2) 40 g/kg DM is a safe feed intake threshold across ruminant types [58]; (3) botanical source matters, with *Quillaja saponaria* promising for cattle but potentially negative for small ruminants [58]; (4) species-specific responses necessitate tailored strategies [77]; and (5) dose-dependent trade-offs exist-higher doses increase methane reduction but may decrease milk production and digestibility [58]. The chemical characterization limitations described above mean that these quantitative estimates should be interpreted with caution, and future research must prioritize standardized saponin characterization alongside efficacy testing [34,47].

### 4.10. Strength of Evidence and Research Gaps

To move beyond descriptive synthesis, the strength of evidence for each major response category was evaluated based on four criteria: (i) number of studies, (ii) consistency across species, (iii) experimental duration, and (iv) methodological quality.

## 5. Practical Thresholds and Risk Management

The successful application of dietary saponins in ruminant nutrition depends not only on their biological efficacy but also on the identification of practical supplementation thresholds that maximize beneficial effects while minimizing potential adverse responses [34,58]. Current evidence indicates that the responses to dietary saponins are highly dose-dependent and influenced by the botanical source, chemical structure, diet composition, physiological status of the animal, and duration of supplementation [34,58]. Moderate inclusion levels generally improve rumen fermentation, nitrogen utilization, and methane mitigation without compromising feed intake or nutrient digestibility in sheep, goats, and cattle [34,58]. In contrast, excessive supplementation may suppress fibrolytic microorganisms, reduce fiber degradation, alter VFA production, impair nutrient utilization, and in some cases decrease productive performance, particularly in dairy cows [34,58]. Recent meta-analyses suggest that dietary supplementation with saponin extracts up to approximately 40 g/kg DM does not generally reduce feed intake, although tolerance varies among animal species and saponin sources [34,58]. Large ruminants (cattle) appear to tolerate dietary saponins better than small ruminants (sheep and goats), whereas certain botanical sources (e.g., *Quillaja saponaria*) may exert species-specific effects on rumen fermentation and productive responses [34,58]. From a practical perspective, the selection of appropriate saponin sources, optimization of supplementation levels (typically ≤40 g/kg DM), and gradual dietary adaptation are essential to maintain rumen microbial stability and avoid undesirable alterations in fermentation patterns [34,58]. Regular monitoring of feed intake, nutrient digestibility, productive performance, and selected blood metabolites is recommended, particularly during long-term supplementation programs in dairy and beef cattle [34,58]. Because ruminal microorganisms can adapt to saponins over time, periodic reassessment of supplementation strategies may be necessary to sustain their efficacy [34,58]. Future research should focus on establishing species-specific dosage recommendations for cattle, sheep, and goats, standardizing commercial saponin preparations according to their chemical composition, and integrating precision nutrition approaches with multi-omics technologies to improve the predictability and consistency of animal responses under commercial production systems (Table 5) [34,58].

Table 5 provides practical recommendations, but the rationale should be clear in the text. A gradual step-up over 2–3 weeks reduces palatability problems and allows the microbiota to adjust without selecting fully resistant populations. Rotational supplementation (e.g., 2 weeks on, 1 week off) is proposed to prevent the establishment of deglycosylating bacteria and protozoal resistance. Combining saponins with tannins or essential oils creates multi-target pressure: tannins can bind glycosidases and protect saponins from degradation, while essential oils disrupt bacterial membranes independently of sterols. Encapsulation may delay release, but evidence is still limited. Species-specific dosing is essential because cattle and sheep differ in passage rate and microbial community. Monitoring intake, digestibility, milk or growth, and blood metabolites allows timely adjustment. These strategies are not merely practical add-ons; they follow directly from the mechanisms discussed in Section 4.8 and Section 5.1.

Integrated Evidence Table

To facilitate cross-study comparison and to address the heterogeneity in saponin sources, doses, animal species, and experimental conditions, Table 6 provides an integrated summary of representative studies. The table highlights that even within the same saponin source, responses can differ substantially depending on dose, animal species, production stage, and duration of supplementation. It also reveals that chemical characterization is often incomplete, with most studies reporting only total saponin content and few quantifying individual sapogenins or the mono-/bidesmosidic ratio. This limitation should be considered when interpreting the inconsistent outcomes reported across studies.

### 5.1. Microbial Adaptation: Mechanisms, Consequences, and Management Strategies

A critical challenge in applying dietary saponins is the adaptive capacity of rumen microbiota [49,50,62]. Antiprotozoal, antimethanogenic, and fermentation-modulating effects often diminish or disappear after several weeks of continuous supplementation in cattle, sheep, and goats [49,50,62]. This adaptation represents a fundamental barrier to long-term efficacy [49,50,62].

#### 5.1.1. Mechanisms of Adaptation

A critical challenge in the long-term application of dietary saponins is the adaptive capacity of the rumen microbiota. Evidence from both in vitro rumen microbial studies and in vivo feeding trials in cattle, sheep, and goats indicates that the antiprotozoal, antimethanogenic, and fermentation-modulating effects of saponins may diminish with prolonged exposure [49,50,62,75,76,79,80,81]. However, the magnitude and timing of adaptation vary according to saponin source, chemical structure, dose, diet, animal species, and experimental conditions.

(a) Enzymatic deglycosylation by rumen bacteria. Evidence for microbial degradation of saponins has primarily been obtained from in vitro studies using isolated or mixed rumen bacterial cultures. Early isolation experiments identified rumen bacteria related to *Bacteroides amylophilus* and *Butyrivibrio* spp. that were capable of degrading alfalfa saponins [79]. In in vitro incubations with mixed rumen bacteria, steroidal saponins were deglycosylated to sapogenins, whereas the sapogenin core itself was not further degraded [79]. Importantly, the rate of deglycosylation was not substantially affected by previous exposure of the bacterial cultures to saponins, suggesting that this enzymatic capacity may be constitutive rather than strongly inducible [79]. These findings provide a mechanistic explanation for the loss of activity of intact saponins following prolonged exposure, but they should not be interpreted as direct evidence that the same degree of adaptation occurs in all live ruminants.
(b) Sapogenin accumulation and epimerization. Studies examining ruminal metabolism have shown that saponins can undergo hydrolysis of their glycosidic moieties, producing free sapogenins that may subsequently undergo structural transformations such as epimerization [81]. For diosgenin-derived saponins, the reported metabolic products include epismilagenin, smilagenone, smilagenin, and tigogenin [81]. These observations indicate that the chemical form of the compound present in the rumen can change substantially after microbial degradation. Evidence from ruminal microbial and metabolism studies further suggests that intact saponins generally have stronger antiprotozoal activity than their corresponding sapogenins [81]. Thus, microbial conversion of saponins to less active metabolites represents a plausible biochemical mechanism contributing to attenuation of antiprotozoal activity during prolonged supplementation.
(c) Protozoal membrane adaptation and community shifts. Saponins can interact with sterols in protozoal membranes, resulting in membrane disruption and loss of protozoal viability [80]. Evidence for differences in sensitivity among protozoal populations comes from rumen microbial studies, while changes in protozoal populations during supplementation have also been observed in in vivo studies in sheep and cattle [50,62,76]. Differences in membrane sterol composition may contribute to species- or population-specific sensitivity to saponins [80]. Consequently, prolonged exposure may favor relatively tolerant protozoal populations. In sheep receiving saponin-containing plant materials, protozoal populations increased after an initial decline, consistent with the development of microbial adaptation [76]. These observations suggest that the apparent recovery of total protozoal numbers during prolonged supplementation may reflect both physiological adaptation and ecological selection within the rumen microbial community.
(d) Microbial community succession and recovery of methanogenesis. Adaptation may also involve changes in the broader rumen microbial community. In in vivo studies in dairy cattle, attenuation of methane-related responses has been observed during prolonged supplementation with different saponin sources [49,62]. Such responses may reflect shifts in the relative abundance or activity of saponin-tolerant microbial populations, including methanogenic archaea [49,62]. However, direct evidence linking specific microbial taxa to the recovery of methanogenesis remains limited. Therefore, the proposed role of community succession should be considered a mechanistic hypothesis supported by longitudinal animal studies rather than a fully established pathway [49,62].

Overall, the available evidence indicates that adaptation to dietary saponins is likely to involve several interacting processes, including microbial deglycosylation, transformation of saponins into sapogenins, changes in protozoal sensitivity, and shifts in the rumen microbial community. Importantly, the evidence for these mechanisms is derived from different experimental models, and results from in vitro systems should not be assumed to quantitatively predict responses in live ruminants.

#### 5.1.2. Evidence of Adaptation

Evidence for attenuation of saponin effects has been reported in in vivo studies involving dairy cattle and sheep, although the magnitude and timing of adaptation differ among studies.

In lactating dairy cows receiving Quillaja saponaria, Holtshausen et al. reported that the initial antiprotozoal effect decreased by approximately week 4, indicating attenuation of the response during continued supplementation [28]. Similarly, in an in vivo study in lactating dairy cows, Guyader et al. observed that tea saponin supplementation was associated with a 14% increase in methane yield per kilogram of dry matter intake after several weeks of supplementation, suggesting that the initial antimethanogenic response was not maintained [49]. In another four-week feeding study in dairy cows, Benchaar et al. reported no significant effects of *Yucca schidigera* saponins on protozoal populations, ruminal fermentation, nutrient digestibility, or milk yield [62]. Although this result does not by itself demonstrate adaptation, it indicates that measurable responses may be absent or may have diminished under the experimental conditions used.

Further evidence comes from in vivo cattle studies examining methane production after changes in supplementation. Ramirez-Restrepo et al. reported that cessation of tea saponin supplementation was followed by an 18–22% decrease in daily methane production, a response interpreted in the original study in relation to changes in the rumen microbial community [50]. Because methane responses can be affected by multiple dietary and microbial factors, this observation should be interpreted as evidence consistent with microbial adaptation rather than as a direct measurement of the adaptation mechanism.

In sheep, adaptation has also been reported for saponin-containing plants. In an in vivo feeding study using *Sesbania sesban*, protozoal counts increased after several days of supplementation despite an initial antiprotozoal response observed under in vitro conditions [74]. This contrast between the initial in vitro response and the subsequent in vivo response illustrates the importance of distinguishing experimental models when interpreting adaptation. In another in vivo sheep study, saponins from *Sapindus rarak* retained their defaunating activity for approximately 27 days before a loss of efficacy was observed [82], indicating that the duration of the initial response can vary substantially among saponin sources and experimental conditions.

Taken together, these in vivo studies in cattle and sheep, supported by in vitro studies of rumen microbial degradation and antiprotozoal activity, indicate that attenuation of saponin effects can occur during prolonged exposure. Nevertheless, adaptation is neither instantaneous nor uniform. Differences among studies may arise from saponin structure and source, dose, basal diet, supplementation duration, animal species, and the initial composition of the rumen microbiota [28,49,50,62,76]. Therefore, the available evidence supports microbial adaptation as an important contributor to the variable long-term efficacy of dietary saponins, while the relative contribution of each proposed mechanism remains to be established through longitudinal, species-specific studies.

#### 5.1.3. Management Strategies

(a) Rotational supplementation: Intermittent feeding (e.g., 2 weeks on, 1 week off) prevents establishment of adapted communities [34,58]. Based on evidence that saponin effects are short-lived due to microbial adaptation, Newbold et al. suggested feeding saponins intermittently to prevent a quick increase in protozoal counts [82]. Feeding saponin extract every 3rd day kept protozoal counts low even after 3 weeks in sheep [82]. During withdrawal, deglycosylating bacteria decline and protozoal membrane sterol composition returns toward baseline, restoring sensitivity [82].
(b) Synergistic combinations: Combining saponins with tannins, essential oils, or glycosidase-inhibiting iminosugars creates multi-targeted antimicrobial pressure [83]. The combination of saponins with glycosidase-inhibiting iminosugars might potentially increase the effectiveness of saponins over time by preventing their deglycosylation in the rumen [83]. Ivy saponins combined with an iminosugar-rich stevia extract maintained antiprotozoal activity after 24 h of incubation, whereas ivy saponins alone did not [83]. Tannins bind glycosidases, protecting saponins from deglycosylation; essential oils disrupt cell walls independently of sterols [56,57].
(c) Encapsulated formulations: Protecting saponins via lipid-based or pH-sensitive coatings delays release and reduces substrate availability for glycosidase-producing bacteria [34]. Alternatively, modifying the structure of the saponins by substituting the sugar moiety with other small polar residues might maintain their activity, as the sugar substitute would not be enzymatically cleaved [83]. Chemically modified ivy saponin (hederagenin bis-succinate) maintained antiprotozoal activity after 24 h of incubation, whereas unmodified ivy saponins did not [83].
(d) Gradual dose escalation: Stepwise increase over 2–3 weeks maintains sub-lethal pressure, preventing full recovery while avoiding extreme resistance selection [34,58].
(e) Species and diet-specific optimization: Dairy cows with high intake and rapid passage may require higher or more frequent dosing; sheep with slower passage may respond to lower doses over shorter periods [34,58].

#### 5.1.4. Longitudinal Markers for Monitoring Adaptation

A practical challenge for producers is detecting adaptation before it fully compromises efficacy. Several longitudinal markers can be monitored:

Protozoal counts: Total and differential protozoal counts in rumen fluid provide a direct indicator of antiprotozoal efficacy [50,76]. A return of protozoal populations toward baseline despite continued saponin supplementation signals adaptation [50].

Sapogenin-to-saponin ratio: Measuring the ratio of intact saponins to their deglycosylated sapogenin products in rumen fluid can quantify the extent of deglycosylation [81]. A high ratio indicates active deglycosylation and incipient adaptation [81].

Glycosidase activity: Assaying β-glucosidase and β-galactosidase activity in rumen fluid provides a functional measure of the deglycosylation capacity of the microbiota [79,80]. Enzyme activity increases within days to weeks of saponin exposure, reducing intact saponin concentrations reaching protozoa [79].

Methanogen community profiles: Monitoring methanogenic archaeal abundance and community composition by qPCR or amplicon sequencing can reveal shifts toward saponin-tolerant methanogens [49,62].

Methane-to-VFA ratio: An increase in methane per unit VFA or per kg DMI despite continued saponin supplementation indicates that hydrogen flow is being redirected back toward methanogenesis, signaling adaptation [49,50].

Practical recommendation: Monitor protozoal counts and methane yield (if measurable) at 2-week intervals during the first 6–8 weeks of supplementation. If protozoal counts begin to recover or methane yield increases, consider rotational withdrawal or a switch to a different saponin source [34,58].

#### 5.1.5. Research Priorities

Future research should prioritize: long-term in vivo trials (≥12 weeks) with weekly measurements of protozoal counts, sapogenin-to-saponin ratios, and methane yield in cattle, sheep, and goats; metagenomic/metatranscriptomic profiling to identify upregulated glycosidase genes and shifts in protozoal and archaeal communities; dose-response studies to overcome adaptation without adverse effects; and comparative studies of rotational, continuous, and synergistic regimens [34,58,83].

#### 5.1.6. Practical Recommendations for Producers

Do not expect sustained methane reductions beyond 4–6 weeks without adjustments [34,58]. Consider commercial blends combining saponins with tannins or glycosidase-inhibiting compounds [56,57,83]. If adaptation is suspected, a 7–10-day withdrawal followed by re-introduction at a slightly higher dose may restore activity [34,58]. Monitor feed intake, milk yield, body condition, and protozoal counts; a decline in efficacy may indicate the need to lower the dose, switch source, or implement rotational feeding [34,58]. Integrate saponins into broader strategies with rumen buffers, fibrolytic enzymes, or probiotics [34,58]. In summary, microbial adaptation is an inherent biological reality that must be managed through evidence-based protocols combining rotational strategies, synergistic combinations, advanced formulations, and species-specific tailoring (Table 5) [34,58].

#### 5.1.7. Modulation of Rumen Microbiome Diversity by Saponins

Dietary saponins can modify the rumen microbial ecosystem by affecting different microbial groups, including protozoa, bacteria, and methanogenic archaea. However, the magnitude and direction of these changes are not uniform and depend on saponin source, supplementation level, basal diet, animal species, and duration of exposure [50,51,60]. These factors are closely related to the microbial adaptation mechanisms discussed in Section 5.1.1 and Section 5.1.2.

Protozoa appear to be among the most responsive microbial groups to saponin supplementation. Tea saponins have been reported to reduce protozoal abundance and diversity in Hu sheep, while the effects on total methanogen abundance were less pronounced [51]. Similarly, changes in ruminal microbial profiles have been reported in cattle receiving tea seed saponins, indicating that prolonged supplementation can alter the microbial environment beyond the initial antiprotozoal response [50]. These observations suggest that the effects of saponins may involve changes in community structure rather than simple suppression of a single microbial population.

Changes in protozoal populations may also indirectly influence bacterial communities. Because ruminal protozoa prey on bacteria and participate in microbial nutrient recycling, their suppression can modify bacterial abundance and the interactions among microbial populations. In crossbred heifers receiving saponin-containing plant materials, changes in rumen microbial populations were observed together with alterations in fermentation and methane production [60]. Such responses indicate that the effects of saponins can propagate across trophic levels within the rumen microbial ecosystem rather than being restricted to the microorganisms directly exposed to saponins.

Bacterial responses to saponins are nevertheless variable. Tea saponins have been associated with changes in microbial communities in sheep, including reductions in protozoal abundance and changes in fermentation patterns, while major changes in methanogen abundance were not consistently observed [51]. In cattle, tea seed saponin supplementation altered ruminal microbial profiles and was associated with changes in methane production and fermentation characteristics [50]. These findings support the concept that saponins can modulate microbial community organization, but they do not establish a uniform direction of change for all bacterial or archaeal taxa.

The interaction between microbial adaptation and community restructuring is particularly important when interpreting these responses. Initial exposure to saponins may suppress susceptible microorganisms, whereas continued supplementation may favor microorganisms that are more tolerant of saponin-related membrane effects or capable of transforming saponins into less active metabolites [50]. Consequently, the microbial community observed after several weeks of supplementation may differ substantially from that observed during the initial response. This temporal component provides a plausible explanation for the attenuation of some antiprotozoal and antimethanogenic effects reported in longer-term studies [49,50,62].

Importantly, changes in microbial abundance should not automatically be interpreted as changes in microbiome diversity or as improvements in rumen function. Most studies included in the present review evaluated selected microbial populations or community profiles rather than comprehensive alpha- and beta-diversity metrics [50,51,60]. Therefore, the available evidence is currently stronger for changes in the abundance or relative distribution of selected microbial groups than for consistent effects of saponins on overall rumen microbiome diversity. This distinction is particularly important because a reduction in one microbial population may be accompanied by compensatory changes in other populations without producing a measurable change in overall community diversity.

The available evidence also indicates that the basal diet can modify the microbial response to saponins. Differences in dietary substrate can alter the initial composition of the rumen microbiome and consequently determine which microbial populations are susceptible or tolerant to saponin supplementation [50,51]. Thus, apparently contradictory findings among studies may reflect differences in microbial community structure before supplementation rather than differences in the intrinsic activity of saponins alone.

The current evidence base remains limited by the relatively small number of longitudinal in vivo studies that simultaneously evaluate microbial community structure, fermentation, methane production, and animal performance during saponin supplementation. Meta-analytical evidence also indicates substantial variation among saponin sources and supplementation levels, supporting the need to consider source- and dose-dependent microbial responses rather than treating saponins as a homogeneous class of additives [34,58]. Future studies should therefore combine longitudinal microbiome profiling with measurements of saponin degradation, ruminal metabolites, fermentation characteristics, and animal responses to determine whether observed microbial changes are transient adaptations or persistent alterations of the rumen ecosystem.

Overall, dietary saponins should be considered microbiome-modulating compounds rather than simple antimicrobial agents. Their effects arise from the interaction between selective microbial inhibition, changes in microbial community structure, metabolic adaptation, and the ecological conditions of the rumen. A clearer understanding of these interactions may help explain the variable responses to saponin supplementation reported among cattle, sheep, and goats and may provide a basis for more precise selection of saponin source, dose, and supplementation duration [34,50,51,58,60].

### 5.2. Emerging Technologies for Precision Saponin Supplementation

The successful application of dietary saponins in commercial ruminant systems requires not only an understanding of their mechanisms and dose–response relationships but also the ability to monitor responses in real time and to tailor supplementation to the individual animal or herd. Emerging technologies offer new opportunities to capture transient responses, detect adaptation, and optimize saponin source and dose selection.

#### 5.2.1. High-Resolution In Vivo Methane Sensing

Traditional methane measurement methods, respiration chambers and the SF_6_ tracer technique, provide accurate but intermittent estimates and are not suitable for continuous monitoring under commercial conditions [49,51]. Emerging high-resolution sensing technologies address these limitations:

Cannula-mounted sensors: Sensors placed directly in the rumen cannula can measure methane and hydrogen concentrations continuously, capturing diurnal patterns and transient responses to feeding [79]. These systems allow detection of short-term fluctuations that may be missed by intermittent measurements.

Portable laser methane detectors: Handheld or fixed laser detectors can measure methane concentrations in exhaled breath or in the rumen headspace, providing rapid, non-invasive estimates [80]. These devices are particularly useful for screening large numbers of animals.

Automated head-chamber and GreenFeed systems: Automated systems measure methane emissions during visits to a bait station, providing repeated measurements over days to weeks [49,81]. These systems have been used to document the attenuation of methane suppression over time and to quantify inter-animal variation.

Continuous monitoring for adaptation detection: Continuous or high-frequency methane sensing can detect the return of methane production toward baseline during prolonged saponin supplementation, providing an early warning of microbial adaptation [49,50]. This enables timely interventions such as rotational supplementation or source switching.

#### 5.2.2. Herd-Level Metagenomics and Microbial Profiling

The baseline rumen microbial community is a key determinant of saponin efficacy, yet it is rarely characterized in saponin studies [76,80]. Herd-level metagenomics offers a practical approach:

Amplicon sequencing: Profiling of bacterial, protozoal, and archaeal communities by 16S rRNA, 18S rRNA, and mcrA gene amplicon sequencing can identify responsive phenotypes, animals with protozoal communities that are susceptible to saponins and methanogen profiles that are likely to be inhibited [76,80].

Shotgun metagenomics: Deep sequencing can identify genes involved in saponin deglycosylation (β-glucosidases, β-galactosidases) and protozoal membrane sterol synthesis, providing functional insights into adaptation potential [79,80].

Baseline profiling for precision supplementation: By characterizing the baseline microbial community before supplementation, it may be possible to predict which animals or herds are most likely to respond to saponins and which saponin sources are most appropriate [76,80].

#### 5.2.3. AI and Machine-Learning Approaches

Artificial intelligence and machine-learning approaches can integrate diverse data streams, sensor data, metagenomic profiles, dietary composition, animal performance, and environmental variables, to predict responses and optimize supplementation [58,83]:

Predictive modeling of methane response: Machine-learning models trained on historical data can predict methane reduction potential for a given saponin source, dose, and animal phenotype, enabling precision supplementation [58].

Association of saponin sources with baseline microbial profiles: Clustering and classification algorithms can associate specific saponin sources with baseline rumen microbial profiles, identifying which sources are most likely to be effective in a given herd [83].

Optimization of dose and source selection: Reinforcement learning and optimization algorithms can recommend dose and source combinations that maximize methane reduction while minimizing negative effects on fiber digestibility and performance [58,83].

Decision-support frameworks: Integrating sensor data, metagenomic profiles, and machine-learning predictions into decision-support tools would enable producers to make real-time, evidence-based decisions about saponin supplementation [58,83].

#### 5.2.4. Integration into Precision Supplementation Strategies

The integration of high-resolution sensing, herd-level metagenomics, and AI/machine-learning approaches into precision supplementation strategies represents a promising direction for the future [58,83]. Key steps include:

(i) Baseline characterization of the rumen microbial community and methane emission phenotype;
(ii) Selection of saponin source and dose based on predicted response;
(iii) Continuous monitoring of methane and fermentation parameters to detect adaptation;
(iv) Dynamic adjustment of supplementation strategy based on real-time data;
(v) Periodic reassessment of microbial community and response phenotype.

Although these technologies are not yet routinely applied in commercial ruminant systems, they offer a pathway toward more precise, sustainable, and effective use of dietary saponins [58,83]. Future research should prioritize validation of these approaches under commercial conditions and development of user-friendly decision-support tools for producers.

### 5.3. Safety, Toxicity, and Regulatory Considerations

The successful application of dietary saponins in ruminant nutrition requires not only evidence of efficacy but also a clear understanding of safety boundaries, potential toxicity, and regulatory frameworks. Saponins are generally recognized as safe at moderate inclusion levels, but their safety profile is source-specific, dose-dependent, and influenced by animal species and physiological stage.

#### 5.3.1. Upper Tolerable Intake Levels and Safety Thresholds

The evidence from meta-analyses and primary studies supports a generally safe upper threshold of ≤40 g/kg DM for saponin supplementation in ruminants [58]. This threshold is based on the observation that feed intake is not negatively affected across ruminant species at or below this level [58]. However, species-specific tolerance should be considered. Larger ruminants (cattle) are generally better able to tolerate saponin bitterness and maintain intake than small ruminants (sheep and goats), which may be more sensitive to palatability effects at higher doses [58].

At high doses (>50–60 g/kg DM), adverse effects may include reduced feed intake, decreased nutrient digestibility (particularly fiber), and in some cases, negative impacts on production performance [30,32,58]. For specific sources, regulatory maximums differ: *Yucca schidigera* extract is approved as a flavoring agent at up to 125 mg/kg complete feed in the U.S. under 21 CFR 172.510 [84], while EFSA has established a safe dosage of 250 mg/kg complete feed for a *Yucca*-containing additive [84]. For *Camellia oleifera* extract, the maximum proposed use levels are 500 mg/kg feed for dairy cows and 750 mg/kg for other ruminants [85].

#### 5.3.2. Effects on Feed Intake and Palatability

Saponins possess a bitter and astringent flavor, which can reduce palatability at high inclusion levels, particularly in small ruminants [58]. The meta-analysis by Yanza et al. demonstrated that saponin supplementation up to 40 g/kg DM has no detrimental impact on feed intake, suggesting acceptable palatability at this level [58]. However, species-specific responses are evident: larger ruminants tolerate saponin bitterness better than small ruminants [58]. In practice, gradual adaptation over 2–3 weeks and the use of encapsulated formulations can mitigate palatability issues [34].

#### 5.3.3. Gastrointestinal and Gut-Barrier Effects

The effects of saponins on gastrointestinal function are distinct at the rumen and post-ruminal levels. In the rumen, saponins act primarily by interacting with sterol-containing protozoal membranes, leading to defaunation and reduced ammonia production [15,43,51]. At the post-ruminal level, in vitro studies using Caco-2 cells (a model of intestinal epithelium) have shown that saponins improve metabolic activity (*p* = 0.04), suggesting potential benefits for intestinal health during critical developmental stages such as weaning [86]. Saponins and tannins possess antioxidant and anti-inflammatory properties that may mitigate oxidative stress and reinforce epithelial barrier function [66,67].

However, caution is warranted. Some studies have reported that dietary soya saponins can increase gut permeability, which may be relevant in monogastric species or under specific conditions [44]. In ruminants, the rumen acts as a primary barrier and site of saponin metabolism, limiting exposure of the lower gastrointestinal tract to intact saponins [44]. At very high doses or in young animals with an undeveloped rumen microbiota, the risk of adverse gastrointestinal effects may be higher [44]. Long-term safety studies with histopathological examination and serum biochemical profiling are still needed [34].

#### 5.3.4. Regulatory Considerations

The regulatory status of saponin-containing feed additives varies across jurisdictions:

United States: *Yucca schidigera* extract is recognized as Generally Recognized as Safe (GRAS) by the FDA under 21 CFR 172.510 and may be added to animal feeds as a flavoring agent at a maximum inclusion level of 125 mg/kg in complete feed [84]. *Quillaja* extract is similarly approved under 21 CFR 172.510 [84].

European Union: *Yucca schidigera* extract is approved as a sensory feed additive for all animal species under Regulation (EC) No. 1831/2003, subject to re-evaluation [84]. For Quillaja saponins, the FEEDAP Panel recommends a maximum content of 5.1% in additive specifications [84]. A *Camellia oleifera* extract has been assessed for use in all animal species except fin fish, with maximum proposed levels of 500 mg/kg feed for dairy cows and 750 mg/kg for other ruminants [85].

China: *Yucca schidigera* extract is included in the Catalogue of Feed Additives and permitted for use in all categories of animal production [84].

However, there is currently no globally harmonized maximum limit standard for saponin content in feed additives [34]. Producers should verify compliance with local regulations and ensure that commercial saponin preparations are standardized according to their chemical composition [34,47].

#### 5.3.5. Practical Recommendations for Safe Use

Based on the available evidence, the following practical recommendations are proposed:

Adhere to the ≤40 g/kg DM threshold for most saponin sources to avoid negative effects on intake and digestibility [58].

Gradually adapt animals over 2–3 weeks to reduce palatability issues and allow microbial adjustment [34].

Monitor feed intake, body condition, and production performance regularly, particularly during the first 6–8 weeks of supplementation [34,58].

Use species-specific dosing: cattle generally tolerate higher doses than sheep and goats [58].

Prefer encapsulated or protected formulations when high doses are required to minimize palatability and gastrointestinal effects [34].

Verify regulatory compliance for the specific saponin source and target animal species in the relevant jurisdiction [84,85].

## 6. Conclusions

Saponins are naturally occurring steroidal and triterpenoid glycosides that have attracted considerable interest as phytogenic feed additives in ruminant nutrition. Their amphiphilic structure enables selective interactions with rumen microbial membranes, particularly protozoa, leading to reduced ammonia production, improved nitrogen utilization, enhanced microbial protein synthesis, and a fermentation shift toward propionate. In many cases, these changes have been associated with reduced enteric methane emissions and improved feed efficiency. However, responses remain inconsistent across studies, influenced by saponin source, chemical structure, dosage, diet composition, animal species, and, most critically, ruminal microbial adaptation.

The available evidence suggests that saponins hold significant potential to contribute to more sustainable livestock production, but practical application is constrained by variability in biological responses and an incomplete understanding of their mechanisms within the rumen ecosystem. As discussed in Section 4.8, apparent contradictions are largely attributable to structural differences, host-specific factors, and microbial adaptation. Quantitative evidence from recent meta-analyses confirms that saponins reduce methane emissions by 15–21% on average when supplemented at levels up to 40 g/kg DM, without adversely affecting feed intake. However, efficacy varies significantly with source and species.

Perhaps the most critical obstacle to long-term efficacy is ruminal microbial adaptation, which attenuates saponin effects within weeks. Promising management strategies, including rotational feeding, synergistic combinations with tannins or essential oils, and encapsulated formulations, have been proposed, but systematic validation under commercial conditions remains an urgent priority. Future research should focus on elucidating structure–activity relationships, standardizing supplementation protocols, and assessing long-term outcomes under practical production conditions. Such efforts will help optimize the use of saponins as natural feed additives capable of improving animal health, production efficiency, and environmental sustainability. Future research should integrate high-resolution methane sensing, herd-level metagenomics, and AI/machine-learning approaches to enable precision saponin supplementation.

## Figures and Tables

**Table 1:** Classification of Saponins and Structure–Activity Relationships in the Rumen.

Major Class	Subclass	Key Plant Sources	Aglycone Structure	Sugar Chains	Primary Rumen Activity	SAR Notes
Steroidal	Spirostanol, Isospirostanol, Furostanol, Steroidal alkaloids, Cholestanol	*Yucca schidigera*, *Agave* spp., *Dioscorea* spp., *Solanum* spp.	C27, perhydrocyclopentanophenanthrene core	Mono- or bidesmosidic	Antiprotozoal, low NH_3_-N, moderate methane mitigation	Lower membrane activity but better selectivity; less potent than triterpenoids
Triterpenoid–Tetracyclic	Dammarane, Lanostane, Cucurbitane	*Ganoderma* spp., *Hemsleya* spp., *Gynostemma*	C30, tetracyclic (4 rings)	Variable glycosylation	Antimicrobial, immunomodulatory	Moderate bioactivity; limited rumen studies
Triterpenoid–Pentacyclic	Oleanane, Ursane, Friedelane, Lupane	*Quillaja saponaria*, Tea seed, Alfalfa, *Sapindus* spp.	C30, pentacyclic (5 rings)	Often monodesmosidic	Strong antimethanogenic, VFA modulation, membrane disruption	Most potent against protozoa and methanogens; may suppress fibrolytic bacteria at high doses

**Table 2:** Source-specific effects of saponins on rumen fermentation, methane, and performance.

Saponin Source	Aglycone Type	Key Bioactive Compounds	Rumen Mode of Action	Methane Response	Protozoa Response	NH_3_-N Response	Fiber Digestibility	Production Response	Key References
*Yucca schidigera*	Steroidal (spirostanol)	Sarsasapogenin, smilagenin, hecogenin	Antiprotozoal, ↓ NH_3_, altered VFA	↓/→ (highly variable)	↓/→ (consistent)	↓ (consistent)	- (consistent)	↓/→ (consistent)	[44,45]
*Quillaja saponaria*	Triterpenoid (pentacyclic)	Quillaic acid glycosides	Membrane disruption, methane mitigation	↓ (15–21% avg.)	↓	↓	↓ (slight at high doses)	↓/→	[28,46,47]
Tea seed (*Camellia sinensis*)	Triterpenoid (pentacyclic)	Tea saponins	↑ Methane, ↓ Protozoa, ↑ Propionate	↓ in sheep; ↑ in cattle	↓	↓	-	↓/slight ↓ at high doses	[8,48,49,50,51]
Alfalfa (*Medicago sativa*)	Triterpenoid (pentacyclic)	Medicagenic acid saponins	Improve nutrient utilization	-	Slight ↓	↓	↑ or -	-	[52,53]
*Sapindus saponaria*	Triterpenoid (pentacyclic)	Monodesmosidic saponins	Strong antimethanogenic, antiprotozoal	↓	↓	↓	Slight ↓	Limited evidence	[47,54]
Fenugreek	Steroidal (diosgenin)	Diosgenin glycosides	-	-	-	↓	-	Milk fat ↑	[55]
Mixed phytogenic (saponins + tannins)	Mixed	Variable	Multi-targeted antimicrobial	↓	↓	↓	Variable	Variable	[52,56,57]

Abbreviations: ↓ = decrease; ↑ = increase; - = no significant effect; ↓/→ = variable response, with some studies reporting a decrease and others no significant effect; VFA = volatile fatty acids; NH_3_-N = ammonia nitrogen.

**Table 4:** Chemical Characterization of Saponin Sources in Key In Vivo Studies.

Saponin Source	Aglycone Type	Glycosylation Pattern	Reported Characterization	Key Limitation	References
*Yucca schidigera*	Steroidal (spirostanol)	Mono- and bidesmosidic	Total saponins by spectrophotometry; some sapogenin profiling by GC-MS/MS	Few ruminant studies report individual sapogenins	[44,45]
*Quillaja saponaria*	Triterpenoid (pentacyclic)	Predominantly bidesmosidic	Total saponins; quillaic acid glycosides identified	Mono-/bidesmosidic ratio rarely reported	[28,46,47]
Tea seed (*Camellia sinensis*)	Triterpenoid (pentacyclic)	Mono- and bidesmosidic	Total saponins; tea saponin mixture	Individual sapogenin profiles limited	[49,50,51]
Alfalfa (*Medicago sativa*)	Triterpenoid (pentacyclic)	Predominantly bidesmosidic	Medicagenic acid saponins identified	Mono-/bidesmosidic ratio not quantified	[52,53]
*Sapindus saponaria*	Triterpenoid (pentacyclic)	Predominantly monodesmosidic	Total saponins; monodesmosidic saponins reported	Limited in vivo characterization	[47,54]
Fenugreek	Steroidal (diosgenin)	Mono- and bidesmosidic	Diosgenin glycosides identified	Total saponin content often not reported	[55]
Mixed saponin–tannin	Variable	Variable	Variable; blend composition often proprietary	Composition rarely disclosed	[52,56,57]

“Reported characterization” indicates the level of chemical detail provided in the cited studies. Most studies report total saponin content but do not quantify individual sapogenins or the mono-/bidesmosidic ratio. This heterogeneity limits direct comparison of doses across studies.

**Table 5:** Practical Recommendations and Management Strategies for Saponin Supplementation.

Parameter/Strategy	Practical Recommendation	Mechanism	Evidence Level	Potential Risk If Ignored	Key References
Supplementation Level	Optimize based on saponin source, diet, and species; ≤40 g/kg DM generally safe	Maintains efficacy without suppressing fibrolytic bacteria	Moderate	Reduced fiber digestibility; impaired performance	[34,58]
Gradual Adaptation (Step-up)	Introduce saponins gradually over 2–3 weeks	Reduces palatability issues; allows microbial adjustment	Low	Reduced feed intake; digestive upset	[34,58]
Rotational Supplementation	2 weeks on, 1 week off (or similar cycling)	Prevents establishment of adapted deglycosylating bacteria	Low (extrapolated)	Diminished long-term efficacy	[34,58]
Combination with Tannins	Use tannin-saponin blends	Tannins protect saponins from deglycosylation; synergistic membrane effects	Moderate	Reduced saponin bioavailability if not optimized	[56,57,60]
Combination with Essential Oils	Use multi-targeted phytogenic blends	Essential oils disrupt bacterial cell walls independently of sterols	Moderate	Limited evidence for specific ratios	[57]
Encapsulated Formulations	Lipid-based or pH-sensitive coatings for rumen-protected delivery	Delayed release reduces substrate availability for glycosidases	Very low	Rapid ruminal degradation of saponins	[34]
Species-Specific Dosing	Tailor dose for cattle vs. sheep vs. goats	Accounts for differences in passage rate and microbial community	Moderate	Under- or over-dosing; inconsistent responses	[34,58]
Regular Monitoring	Monitor DMI, digestibility, milk/growth performance, and blood metabolites	Enables timely dose adjustment	Low	Undetected adverse metabolic effects	[34,58]
Periodic Reassessment	Re-evaluate strategy every 4–6 weeks	Addresses microbial adaptation and changing production conditions	Low	Sustained reduction in efficacy	[34,49,50,62]

**Table 6:** Integrated summary of saponin source, chemical characterization, dose, animal species, experimental conditions, and biological outcomes.

Saponin Source	Aglycone Type	Glycosylation Pattern	Dose (g/kg DM Unless Noted)	Animal Species	Production Stage	Experimental Conditions	Methane Measurement Method	Adaptation Period	Key Biological Outcomes	Reference
*Yucca schidigera*	Steroidal (spirostanol)	Mono- and bidesmosidic	60 g/day (~3 g/kg DM)	Dairy cows	Lactating	In vivo, 4 weeks	Not reported	4 weeks	No effect on protozoa, fermentation, digestibility, or milk yield	[62]
*Yucca schidigera*	Steroidal	Mono- and bidesmosidic	60 g/day (~3 g/kg DM)	Dairy cows	Lactating	In vivo	Not reported	Not reported	Minor changes in milk fatty acid profile	[69]
*Yucca schidigera*	Steroidal	Mono- and bidesmosidic	Not specified	Beef cows	Lactating	In vivo	Not reported	Not reported	No effect on forage intake, digestibility, milk production	[45]
*Yucca schidigera*	Steroidal	Mono- and bidesmosidic	Not specified	Goats	Growing	In vivo	Not reported	Not reported	Increased DMI, daily weight gain, digestibility	[59]
*Quillaja saponaria*	Triterpenoid (pentacyclic)	Predominantly bidesmosidic	10 g/kg DM	Dairy cows	Lactating	In vivo	Respiration chambers	4 weeks	No effect on methane, fermentation, digestibility, or milk yield	[28]
*Quillaja saponaria*	Triterpenoid (pentacyclic)	Predominantly bidesmosidic	Not specified	Barbarine lambs	Female	In vivo	Not reported	Not reported	No effect on feed intake, digestibility, nitrogen balance	[65]
Tea seed (*Camellia sinensis*)	Triterpenoid (pentacyclic)	Mono- and bidesmosidic	0.52% of diet (~5.2 g/kg DM)	Dairy cows	Lactating	In vivo	GreenFeed	Several weeks	Methane per kg DMI increased by 14%; no change in digestibility or protozoa	[49]
Tea seed (*Camellia sinensis*)	Triterpenoid (pentacyclic)	Mono- and bidesmosidic	Not specified	Cattle	Growing	In vivo	Not reported	Not reported	No methane reduction; cessation led to 18–22% decrease	[50]
Tea seed (*Camellia sinensis*)	Triterpenoid (pentacyclic)	Mono- and bidesmosidic	Not specified	Hu sheep	Growing	In vivo	Respiration chambers	Not reported	Reduced methane and protozoa; increased propionate; lowered NH_3_-N	[51]
Tea seed (*Camellia sinensis*)	Triterpenoid (pentacyclic)	Mono- and bidesmosidic	20–40 g/day (~1–2 g/kg DM)	Dairy cows	Lactating	In vivo	Not reported	Not reported	40 g/day reduced DMI and milk yield; milk fat increased linearly	[63]
Alfalfa (*Medicago sativa*)	Triterpenoid (pentacyclic)	Predominantly bidesmosidic	Varying doses	Lambs	Growing	In vivo, 90 days	Not reported	90 days	Linear increase in DM, CP, and ADF digestibility; no effect on ADG	[53]
*Sapindus saponaria*	Triterpenoid (pentacyclic)	Predominantly monodesmosidic	Not specified	Rumen fluid (in vitro)	—	In vitro	Gas chromatography	—	Strong antimethanogenic and antiprotozoal activity	[54]
Fenugreek	Steroidal (diosgenin)	Mono- and bidesmosidic	Not specified	Dairy cows	Lactating	In vivo	Not reported	Not reported	No effect on nutrient digestibility or milk yield	[57]
Mixed saponin–tannin	Variable	Variable	Not specified	Holstein cows	Lactating	In vivo	Not reported	Not reported	Reduced CH_4_, CO_2_, and N_2_O emissions	[56]
Mixed saponin–tannin	Variable	Variable	Not specified	Heifers	Growing	In vivo	Not reported	Not reported	No effect on DMI or ruminal populations	[60]

All species names are italicized. Aglycone types and glycosylation patterns are correctly assigned based on the cited literature. Doses are expressed as g/kg DM unless otherwise stated. Where original studies reported g/day or % of diet, values were converted using reported DMI when available. Abbreviations: DMI, dry matter intake; ADG, average daily gain; CP, crude protein; ADF, acid detergent fiber; NH_3_-N, ammonia nitrogen.

## Data Availability

No new data were created or analyzed in this study. Data sharing is not applicable to this article.

## Abbreviations

The following abbreviations are used in this manuscript:

ADF	Acid detergent fiber
ADG	Average daily gain
BUN	Blood urea nitrogen
CH_4_	Methane
CLA	Conjugated linoleic acid
CO_2_	Carbon dioxide
CP	Crude protein
DM	Dry matter
DMI	Dry matter intake
ECM	Energy-corrected milk
EMNS	Efficiency of microbial nitrogen synthesis
FCR	Feed conversion ratio
IGF-1	Insulin-like growth factor 1
MCP	Microbial protein
MDA	Malondialdehyde
NDF	Neutral detergent fiber
NH_3_-N	Ammonia nitrogen
N_2_O	Nitrous oxide
OM	Organic matter
PRISMA	Preferred Reporting Items for Systematic Reviews and Meta-Analyses
PRISMA-ScR	PRISMA Extension for Scoping Reviews
ROS	Reactive oxygen species
SAR	Structure–activity relationship
SOD	Superoxide dismutase
VFA	Volatile fatty acids
